# Dynamic In‐Plane Heterogeneous and Inverted Response of Graphite to Fast Charging and Discharging Conditions in Lithium‐Ion Pouch Cells

**DOI:** 10.1002/smsc.202200067

**Published:** 2023-04-19

**Authors:** Abigale P. Monasterial, Peter J. Weddle, Kristen Atkinson, David S. Wragg, Andrew M. Colclasure, Francois L. E. Usseglio-Viretta, Natalie Seitzman, Jun-Sang Park, Jonathan Almer, Kandler Smith, Donal P. Finegan

**Affiliations:** ^1^ National Renewable Energy Laboratory 15013 Denver West Parkway Golden CO 80401 USA; ^2^ Centre for Materials and Nanotechnology University of Oslo 0315 Oslo Norway; ^3^ Department of Chemistry Colorado School of Mines 1500 Illinois Street Golden CO 80401 USA; ^4^ X-ray Science Division Advanced Photon Source Argonne National Laboratory Lemont IL 60439 USA

**Keywords:** fast charging, heterogeneity, Li-ion battery, modeling, X-ray diffraction

## Abstract

Solutions for improving fast charging of lithium‐ion batteries have largely focused on alleviating through‐plane lithiation gradients while little is understood about in‐plane heterogeneities and how to resolve them. Herein, high‐speed synchrotron X‐ray diffraction (XRD) resolves graphite lithiation spatially and temporally during 6 C charging and 2 C discharging. At every point during operation, considerable differences in the state of lithiation across the pouch cell are present. Some regions are more responsive to operation than others, reaching full lithiation early during charge and full delithiation during discharge. Other regions within the cell never fully delithiate during discharge, despite a prolonged voltage hold at 2.8 V. Using time‐resolved XRD data, the calculated local current density (mA cm^−2^) at the graphite surface shows an unexpected occurrence of local inverted current densities where regions of graphite are observed to delithiate during charging and lithiate during discharging. A pseudo‐3D model is developed for the graphite electrode with spatially varying microstructural tortuosity to show how microstructural heterogeneity could influence spatial charge dynamics. The model could not predict the complex in‐plane charge behavior observed within the cell. Consequently physics‐based charging protocols based on homogeneous electrode assumptions may underestimate the local variations in charge dynamics and occurrence of lithium plating.

## Graphical abstract

It is becoming increasingly apparent that the local electrochemical response of active materials within Li‐ion batteries is very heterogeneous, yet little is understood about spatial heterogeneity of graphite's response during fast charging. This work applies high‐speed synchrotron X‐ray diffraction to resolve graphite lithiation spatially and temporally during 6 C charging and 2 C discharging.

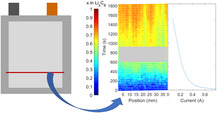

## 1 Introduction

Demand is rapidly growing for light duty electric vehicles (EVs) that rely almost entirely on lithium (Li)‐ion batteries for energy storage. Enabling EVs to charge quickly, to 80% state of charge in 10 min, without sacrificing the cycle life and energy density of the cell, would vastly accelerate the uptake of EVs. However, as the graphite electrode lithiates during charging, there is a limit to the applied overpotential before Li plates on the surface of the graphite instead of intercalating. If the surface potential of graphite falls below the equilibrium potential of 0 V vs Li/Li+, Li plating can occur.^[^ ^1^ ^]^ This condition is met at high rates of charge and since Li plating accelerates capacity fade in cells,^[^ ^2^ ^]^ it is one of the major limitations to fast charging of EVs.

In almost every cell, Li plating occurs unevenly across an electrode as demonstrated by X‐ray diffraction (XRD)^[^ ^3^, ^4^, ^5^, ^6^ ^]^ of fast‐charged pouch cells, optical microscopy of graphite electrodes during high‐rate lithiation,^[^ ^7^ ^]^ and through posttest photographs after dissassembly.^[^ ^2^, ^8^ ^]^ For a given electrolyte, the propensity of a graphite electrode to incur plating is strongly influenced by the electrode's microstructure.^[^ ^9^ ^]^ During fast charging, severe Li concentration gradients in the electrolyte along the through‐plane direction can lead to high currents being experienced by the graphite near the separator.^[^ ^10^, ^11^, ^12^, ^13^ ^]^ Li concentration gradients are particularly severe for electrodes with high tortuosity and thicknesses due to the increased ionic transport resistance.^[^ ^8^, ^14^ ^]^ During high‐rate charging, such lithiation gradients can lead to Li plating near the separator due to the local high current density on the graphite, local solid‐state transport limitations, and consequent drop in potential to below 0 V vs Li/Li+.^[^ ^11^ ^]^ Extensive research efforts have focused on reducing through‐plane heterogeneities by improving electrolyte properties for fast Li transport^[^ ^8^ ^]^ or by reducing electrode tortuosity,^[^ ^15^, ^16^ ^]^ but less is understood about the magnitude and causes of in‐plane heterogeneities in cells and solutions for reducing such heterogeneity.

In‐plane heterogeneities in the microstructure of electrodes as well as particle properties are likely to influence the response of graphite and risk of Li plating nucleation locally throughout a given cell. Previous in‐plane profiling work by Okasinski et al.^[^ ^17^ ^]^ showed that in coin cells, uneven compression of the polymer separator can lead to spatial variations in resistivity. Slight variations in compression are insignificant at low rates but can make profound differences to the local propensity to incur lithium plating at high rates. Defects in separators can also lead to heterogeneous current densities across electrodes and localized lithium plating.^[^ ^18^ ^]^ Individual particles within an electrode can respond differently depending on their morphology and crystallographic properties,^[^ ^19^, ^20^ ^]^ which may lead to some particles incurring higher current densities than others even for a homogeneous electrode microstructure. It is still not well understood how heterogeneous the response of graphite is within commercial cells under fast charging conditions and why Li plating occurs in some regions more than others. Yet, it is critical to understand not only the overall response of a specific cell^[^ ^21^ ^]^ but also the local responses within the cell to tailor optimal fast charging protocols that avoid the occurrence of Li plating. Fast‐charging protocols that are determined by modeling or even a combination of modeling and experiments^[^ ^2^, ^22^, ^23^ ^]^ may benefit from understanding the extent of heterogeneous response of graphite within the cells and thus to adjust the protocol accordingly. Data‐driven methods^[^ ^23^ ^]^ and data‐driven methods combined with physics‐based modeling may be more effective for accommodating heterogeneities since they are largely determined by experimental data on battery degradation that would reflect regional plating accruing from heterogeneities; however, there may still be opportunities for further improving fast charging capabilities by understanding and improving the extent of heterogeneous response within cells.

In this work, high‐speed operando XRD is applied in plane across a pouch cell to quantify the spatial response of graphite to fast charging conditions. Measurements are taken at 0.5 s intervals and 1 mm step sizes across 38 mm of a single‐layer pouch cell. The spatial dynamics of lithiation and delithiation, as well as local current densities, are measured and compared to multiphysics models to help explain the causes of the spatially dependent graphite response.

## 2 Experimental Section

### 2.1 Cell Details and Cycling Conditions

Single‐layer pouch cells of 130 mAh capacity were made with dimensions of 45 mm × 58 mm. The positive electrode consisted of 90 wt% ECOPRO LiNi_0.6_Mn_0.2_Co_0.2_O_2_ (NMC622) with 5 wt% Timcal C45 and 5 wt% Solvay 5130 polyvinylidene fluoride (PVDF). In its dry state, the coating thickness was 112 μm on a 20 μm thick aluminum foil. The coating loading was 30.24 mg cm^−2^ with a density of 2.70 g cm^−3^ and a porosity of 34%. The areal capacity of the electrode was 4.60 mAh cm^−2^ at 0.1 C between 2.8 and 4.2 V vs graphite. The diameters for particle size distribution were D10 = 8.4 μm, D50 = 11.0 μm, and D90 = 14.4 μm. The negative electrode consisted of 91.83 wt% Superior graphite SLC1520P with 2 wt% Timcal C45, 6 wt% Kureha 9300 PVDF Binder, and 0.17 wt% oxalic acid. In its dry state, the coating thickness was 101 μm on a 15 μm thick copper foil. The coating loading was 13.97 mg cm^−2^ with a density of 1.38 g cm^−3^ and porosity of 36.2% with an areal capacity of ≈4.2 mAh cm^−2^. The mass (sieve) based diameters for particle size distribution were D10 = 11.03 μm, D50 = 16.94 μm, and D90 = 26.76 μm. An N/P ratio of 0.91 was used to ensure full lithiation of graphite and potentially Li plating would occur.

During the experiment, the cell was cycled at 6 C (0.78 A) from 2.8 to 4.2 V at constant current (CC) and then held at constant voltage (CV) for 25 min. Cells were discharged at 2 C (0.26 A) at CC from 4.2 to 2.8 V and held at CV for 25 min. Long CV holds were used to ensure cell was fully charged or discharged.

### 2.2 Synchrotron X‐ray Imaging Conditions

**Figure 1:**
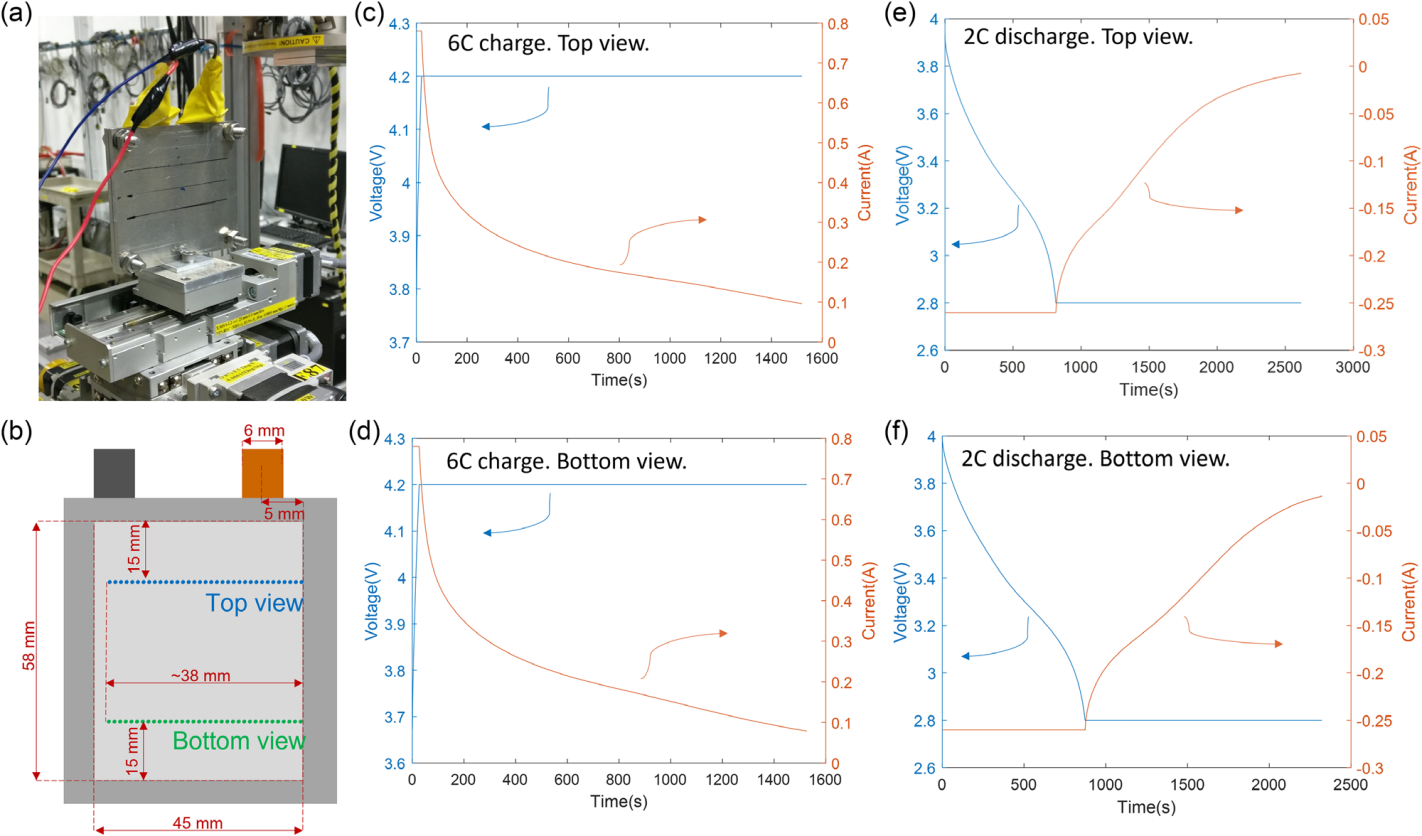
(a) Photograph showing the pouch cell clamped between aluminum plates during the experiment. (b) Dimensions of the pouch cell and the internal electrodes (light gray) with position of top and bottom line scans. c–f) 6 C charge and 2 C discharge cycles for the respective top and bottom views, in order of occurrence.

The experiment was conducted at beamline 1‐ID at the Advanced Photon Source (APS). A 71.68 keV beam of 200 μm × 200 μm was used with a Dexela 2923 CMOS flat panel detector with a square pixel pitch of 75 μm. The distance from sample to the detector was 2080 mm. The synchrotron X‐ray measurement used a transmission geometry with the incident beam impinging on the center of the area detector (protected by a beam stop).^[^ ^24^ ^]^ The pouch cell was clamped between two 2 mm thick aluminum plates that were bolted together (finger tight plus a half turn) as shown in **Figure** **1**a. Please note that the pressure exerted on the cell was not quantified for this work but may have influenced the behavior of the cell described in this manuscript. The cell was moved horizontally across the sight of the beam in 46 steps with 1 mm between each step. The cell was positioned such that the starting point was within the cell, and the final point was outside the cell, thus recording data across the edges of the electrodes. The exposure time was 0.4 s for each measurement. It took ≈20 s to complete each line scan of measurements and ≈17 s to return to the beginning for the next line scan. This process was repeated 50 times for each cycle, recording around 30 min of data each time. For each consecutive cycle, the cell was moved between two vertical positions as shown in Figure 1b.

### 2.3 Data Processing and Rietveld Refinement

**Figure 2:**
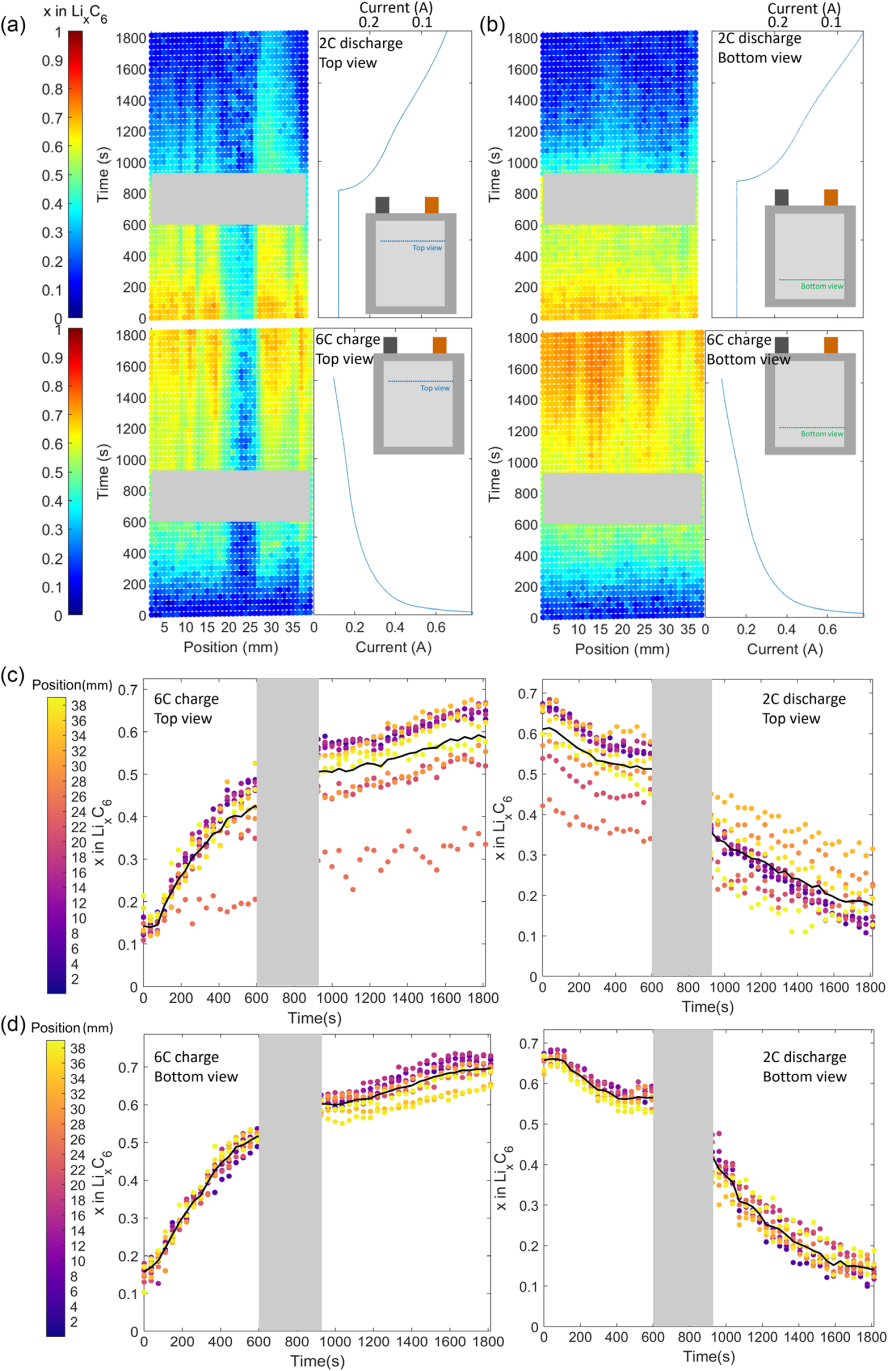
The state of lithiation *x* in Li_x_C_6_ spatially and temporally measured within the pouch cell during 6 C charge and 2 C discharge measured at a) the top view and b) the bottom view, alongside the current measured from the potentiostat. c,d) Corresponding plots of *x* in Li_*x*_C_6_ during charge and discharge. The grayed regions are where disruptions in the measurement occurred and the data were not reliable.

**Figure 3:**
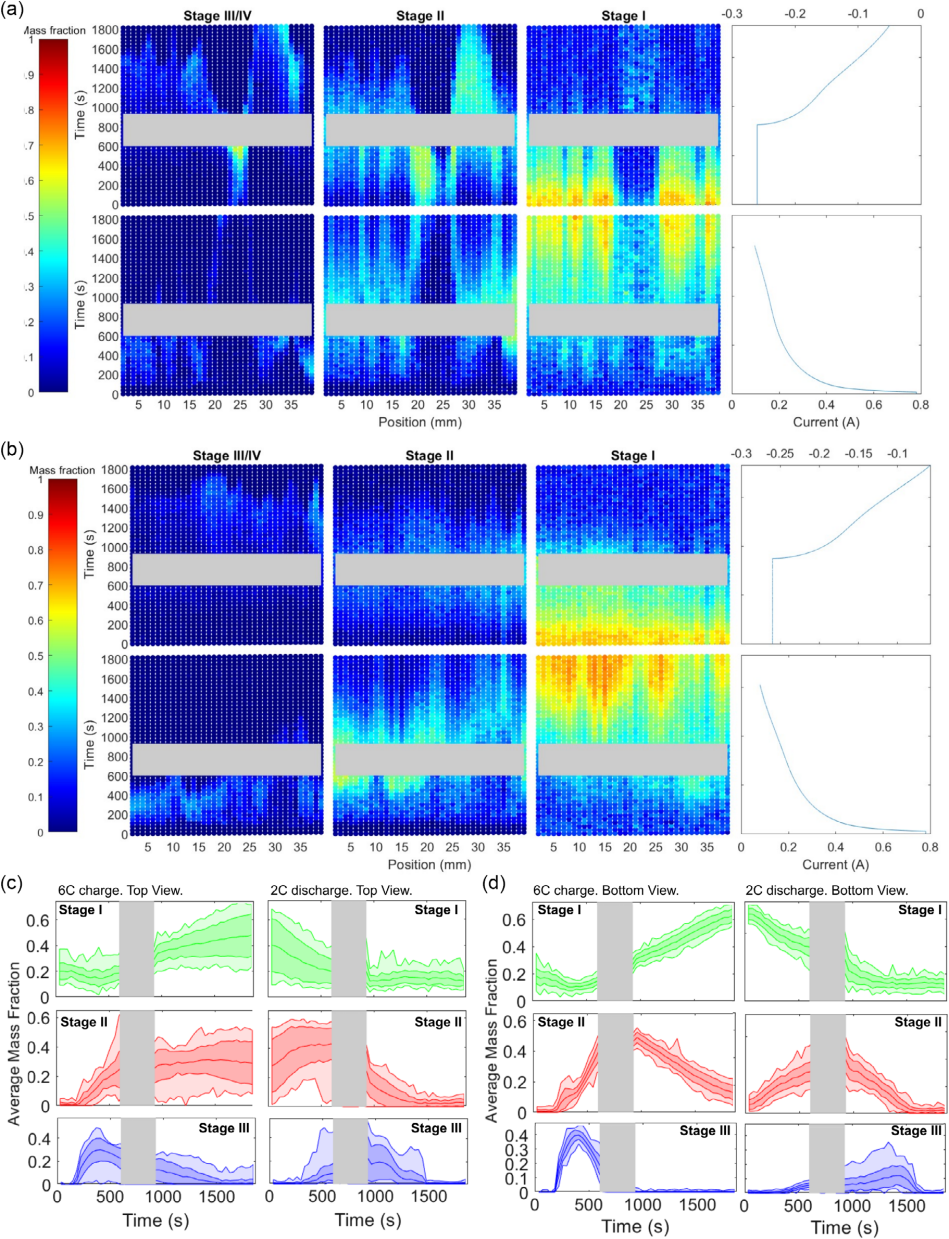
Spatial and temporally resolved mass fractions of the three lithiation stages during the 6 C charge step (bottom row) and subsequent 2 C discharge step (top row) for a) the top view of the cell and b) the bottom view of the cell. Plots showing the averaged evolution of each phase fraction with dark bounds indicating one standard deviation from the mean, and light bounds indicate the maximum and minimum quantities at each time for c) the top view, and d) the bottom view. The grayed regions are where disruptions in the measurement occurred and the data were not reliable.

The XRD data were fitted using TOPAS V6,^[^ ^25^ ^]^ using a surface Rietveld refinement^[^ ^26^ ^]^ approach to process blocks of about 1000 diffraction patterns from several points in time and space simultaneously. To process the large amount of data efficiently, the surface refinement blocks were run sequentially using an MS‐DOS batch file after an initial check that the fitting proceeded in a reasonable manner. A second visual check of selected fits was made after the batch processing to ensure that all the data were being fitted in a meaningful manner, Figure S1, Supporting Information, shows 2D time/space maps of Rwp (the fit quality parameter minimized in Rietveld refinement) for both cells during charge and discharge. The maps are largely flat with Rwp around 6, indicating that the fit is of good quality at all points and that there is no correlation between the fit quality and the features observed in the graphite structure‐based maps shown in **Figure** **2** and **3**. Example Rietveld fits for both cells during charge and discharge are shown in supporting Figure S2, Supporting Information. The first and last 6–7 points of each line scan were not analyzed, as the XRD signal was noisy due to the beam running off the edges of the sample. As the beam passes through the entire stack each point included data from the graphite anode. The graphite was fitted by the Rietveld method (str phases in TOPAS), while the other components were fitted using individual peaks with refined position, intensity, and broadening (pks phases in TOPAS). The data range only includes one or two peaks for each possible graphite phase, and all of the peaks are of the (0 0 l) Miller class, which provides information about the *c*‐axis only. The structures used for graphite phases are all hexagonal, and so the *a/b* axes (equal by symmetry) were fixed to literature values, and the *c*‐axes were refined with parameter limits to stop one phase fitting peaks which actually correspond to other structures (very common for the gradual lattice parameter changes between the graphite stages). In all, 36 parameters were refined for each diffraction pattern: *c*‐axis, Lorentzian size broadening, and scale for the five Li_*x*_C_6_ phases and two NMC phases; position, area, and peak width for each of three broad peaks attributed to poorly crystalline materials like the electrolyte and separator and a 6‐term Chebyshev polynomial background. An example of the input TOPAS file is included in Supplementary information. Zero‐point errors and sample to detector distance variations for the different battery components were ignored. Starting structures were obtained from the ICSD PDF database. To rationalize the calculations in the surface refinements, “conserve_memory” and “approximate_A” commands were applied in the input file. Parameter errors were obtained using bootstrap methods.

### 2.4 Estimating Lithiation and Current Density

During charging, graphite undergoes distinct lithiation stages. These stages describe the average number of graphene layers between each Li layer, e.g., Stage II refers to LiC_12_ where there are two graphene layers between each Li layer, on average. Similarly, Stage III refers to LiC_18_ and Stage I to LiC_6_. Using the mass fractions of each phase determined by Rietveld refinement, we estimated lithiation within the electrode by summing the measured stages of lithiation. It should be noted that fitting intermediate phases between graphite and LiC_12_ were challenging, but a sensitivity analysis in previous work^[^ ^11^ ^]^ showed that due to the low‐ and short‐lived presence of these phases, there contribution to the overall lithiation state of the electrodes is expected to be minimal. Stage IV, a very low lithium content solid solution, as described by Dahn in 1991,^[^ ^27^ ^]^ was included in the fitting but is calculated as graphite for the later calculations (see Supporting Information for plots of the distribution of graphite and stage IV from different Rietveld models) as its crystal structure is not known and its lithium content is minimal. It was also assumed that the graphite anode evenly maintained its loading values (13.97 mg cm^−2^ with a density of 1.38 g cm^−3^ and porosity of 36.2% with an areal capacity of ≈4.2 mAh cm^−2^). We thereafter calculated local current densities by the change in Li_*x*_C_6_ measured over time. Equations outlining this approach are provided in Supplementary Information and are similar to an approach used and described previously.^[^ ^11^ ^]^

### 2.5 Multiphysics Modeling

The pseudo‐3D (P3D) model is developed in COMSOL multiphysics, with parameters and equations reported in the Supporting Information. To induce in‐plane heterogenous utilization, the electrolyte tortuosity is assumed to vary in accordance with work from Liu et al.^[^ ^28^ ^]^ and details are shown in the Supporting Information. This heterogeneous tortuosity is implemented in both the cathode and anode domains. This model predicts in‐plane and through‐plane Li intercalation heterogeneities due to electrolyte transport alone. In the physical system, there are additional sources of heterogeneities such as Li‐plating nucleation,^[^ ^29^, ^30^ ^]^ resultant graphite inactive “shadow,”^[^ ^31^ ^]^ solid‐phase staging barriers,^[^ ^32^, ^33^ ^]^ and particle grain orientation.^[^ ^34^ ^]^ The P3D model results illustrate expected ranges of heterogeneities induced by electrolyte transport.

## 3 Results

### 3.1 Electrochemical Data

To investigate the heterogeneous behavior of the graphite electrode during fast charging, we used XRD to nondestructively measure lithiation during operation of a fully assembled pouch cell. 46 XRD point measurements 1 mm apart were taken in‐plane across the cell at the top and bottom of the cell 15 mm from the respective edge (Figure 1a,b), to provide insight on how the behavior of graphite changed across the surface of the cell. Since the final few points of each line scan crossed the edge of the electrode, the in‐plane distance of electrode covered was ≈38 mm. The tabs were located 5 mm inward from the cell's edges and were 6 mm wide. In the rest of the manuscript, the aluminum tab is in line with in‐plane positions 3–9 mm, while the copper tab is in line with positions 32–38 mm. The cell was cycled between 2.8 and 4.2 V for two consecutive cycles at 6 C CC charge followed by CV for 25 min and a 2 C CC discharge followed by CV for 25 min. A 30 min rest occurred between charge and discharge. The current and voltage data for the cycles are shown in Figure 1c‐f and show that the 6 C CC step was achieved for about 25 s and the 2 C CC step for about 730 s in each case. Using identical cell materials, it was observed in previous work that Li plating occurred during the 6 C conditions.^[^ ^11^ ^]^

### 3.2 Lithiation Heterogeneity

As shown in Figure 1, XRD measurements were recorded along lines located near the top and the bottom of the cell. For each measurement, the lithiation state of the graphite, *x* in Li_*x*_C_6_, was determined from the mass fractions of the individual lithiation stages, as described in the methods section. In Figure 2a–d, the lithiation state for each position over time is shown as a colormap for the 6 C charge steps as well as the 2 C discharge steps. The grayed regions are where disruptions in the measurement occurred and the data were not reliable. The lithiation map shown for the top position in Figure 2a shows that for a width of about 6 mm between position 20 and 26 mm, there was very little response of the graphite to the charge or discharge conditions. The cause of this is unknown but may have occurred due to phenomena like a local gas pocket, locally overcompressed separator with constricted pores, or poor electrical connection of the electrode to the current collector. The difference between the maximum and minimum values for *x* in Li_*x*_C_6_ along the measured region of the top view was 0.3 (between 0.35 and 0.65 as shown in Figure 2c).

The graphite at the region near the bottom of the cell displayed a more homogeneous response to the charging and discharging conditions, as seen in Figure 2b. However, despite the more homogeneous response, there was still a difference in *x* of 0.1 across the measured region, between an *x* of 0.6 and 0.7. For both positions during 6 and 2 C charge, the rate of divergence of *x* was greatest after the cell reached an average *x* of 0.4. This is likely to be influenced by the free energy barrier associated with the transition from Stage II (LiC_12_) to Stage I (LiC_6_) and will be discussed in more detail in the following section that looks at the evolution of distinct lithiation stages. On discharge, the top view showed significant differences in the local rates of delithiation with the regions of initial highest *x* delithiating fastest. Upon discharge some regions showed an *x* of greater than 0.2, even after 1500 s. Residual lithiated graphite was observed in previous work where lithium plating occurred and lithiated phase was shown to consist primarily of Stage I^[^ ^11^ ^]^; this will be explored in greater detail in the following section.

Figure S8, Supporting Information, reproduces the same data as Figure 2c,d, but instead with respect to cumulative capacity as opposed to with respect to time. If the graphite intercalates homogeneously across the cell, the average graphite intercalation fractions will increase/decrease linearly when plotted with respect to capacity. During the beginning of charge, the data show a linearly increasing intercalation fraction along both the top and bottom view. However, near the end of charge, the slope decreases, indicating that less Li is intercalated into the graphite per unit charge transferred. Additionally, at the beginning of discharge, the data indicate that the average intercalation fraction is relatively constant. A relatively constant intercalation fraction means that current is being sourced from somewhere else, such as from side reactions from Li plating being stripped during the first half of discharge as opposed to deintercalating from the graphite. The potentiostat measured ≈90 mAh cumulative capacity (see Figure S8, Supporting Information). With an active area of 26.1 cm^2^, a theoretical capacity of 4.2 mAh cm^−2^, and a starting intercalation fraction of *x*_init_ ≈ 0.15 (see Figure 2c,d), if all of the current resulted in intercalation, the average intercalation fraction at the end of charge would be *x*_final_ ≈ 1.06. This is above the theoretical limit. Therefore, some current must come from either Li plating that occurred during charging or from side reactions. The XRD data did not show any sign of Li plating, but we acknowledge that some Li plating may have been below the detection limit of the technique. The final average intercalation fraction is measured to be *x*_final_ ≈ 0.6 (see Figure 2c,d), which indicates that ≈50 mAh of the charge resulted in graphite lithiation.

### 3.3 Graphite Lithiation Staging Analysis

Color maps in Figure 3 show the local evolution of mass fractions of Stage III, Stage II, and Stage I during lithiation and delithiation of the graphite. Figure 3a shows the top view, and Figure 3b shows the bottom view of the 6 charge and 2 C discharge processes. During charge, intercalation transitions from Stage III to Stage II and to Stage I, as seen by the appearance and disappearance of Stage III, then Stage II, and finally the appearance of Stage I. The regions with highest lithiation in the top view reached around *x* = 0.7, while the region with the low response between positions 20 mm and 25 mm only reached about *x* = 0.3 by 1800 s into the charge step. The region within the bottom view of the cell displayed a faster and more homogeneous lithiation response during charge, as shown in Figure 3b. Stage III appeared in the bottom view before the top view and the region in the bottom view more quickly transitioned to Stage II and subsequently to Stage I. The final composition of Stage I in the bottom view was higher than the top view, reaching around *x* = 0.8. Unexpectedly, the less active region in the top view showed a mass fraction of around 0.3 of Stage I after 1800 s, but did not experience the progression of Stage III and Stage II before getting to Stage I; this may be indicative of Li plating occurring around that region which due to the low detection limits of Li may not have been apparent in the XRD data.^[^ ^35^ ^]^ The respective evolution of lithiation stages for the top and bottom views during charge is plotted more clearly in Figure 3c‐d, where the limits of the semi‐transparent overlays on the average value represent the maximum and minimum lithiation values along those lines.

On discharge, the inverse stage sequence was observed, starting with Stage I, and progressing through Stage II and Stage III toward graphite. The top view showed a highly heterogenous sequence of staging from Stage I toward graphite. The low‐activity region contained residual Stage I by the end of discharge with a mass fraction of around 0.3. This is consistent with previous work^[^ ^11^ ^]^ where LiC_6_ was observed following discharge where Li plating was present. To the right of the low‐activity region, Stage II appeared after transition from Stage I and maintained a high presence for over 1000 s before quickly transitioning through Stage III. It is not known what caused this prolonged period on Stage II, but one explanation is that the local overpotential was not sufficient to complete the delithiation transition to Stage III and graphite. The length of time for which Stage III existed at each position was consistently much less than Stage II, which may also be explained by the relatively low change in Gibbs free energy between Stage II and graphite than between Stage I and Stage II.^[^ ^36^ ^]^ In previous work,^[^ ^11^ ^]^ the local rate of lithiation was shown to be influenced by the graphite stages already present. For example, when the graphite nearest the separator reached Stage II, further lithiation shifted to regions deeper into the electrode before returning to the graphite nearest the separator to lithiate to Stage I; under some conditions, it may be electrochemically favorable to lithiate elsewhere due to there not being a sufficient overpotential to bridge the free energy barrier associated with phase transitions. This phenomenon may also have consequences on in‐plane lithiation too as will be explored in the following section on current density.

At the bottom view region, the transition from Stage I, to Stage II, and Stage III was much more homogeneous. Stage II existed for a longer period of time but was generally a lower mass fraction than the top view, and while Stages II and III reached close to 0% mass fraction by 1800 s, residual Stage I amounting to a mass fraction of around 0.3 remained.

### 3.4 In‐Plane Heterogeneous Current Density

The rate of change of *x* in Li_*x*_C_6_ for each position within the cell was used to estimate the current density per unit surface area of graphite. This will be referred to as “current density” in mA cm^−2^ of graphite surface and is different from the current per unit surface area of the current collector, which is also expressed with the same units. The current density was calculated by using the specific surface area of the graphite that was provided by the manufacturer (0.89 m^2^ g^−1^) and the mass of graphite expected to be within the field of view during the XRD measurement. The equations used to calculate the current density are taken from previous work^[^ ^11^ ^]^ and are provided in Supporting Information. The values for current density should be considered with some caveats: it is assumed that the current is homogeneous throughout the depth of the electrode, that the specific surface area value of 0.89 m^2^ g^−1^ is accurate for all regions, and that all surface area is equally used.

**Figure 4:**
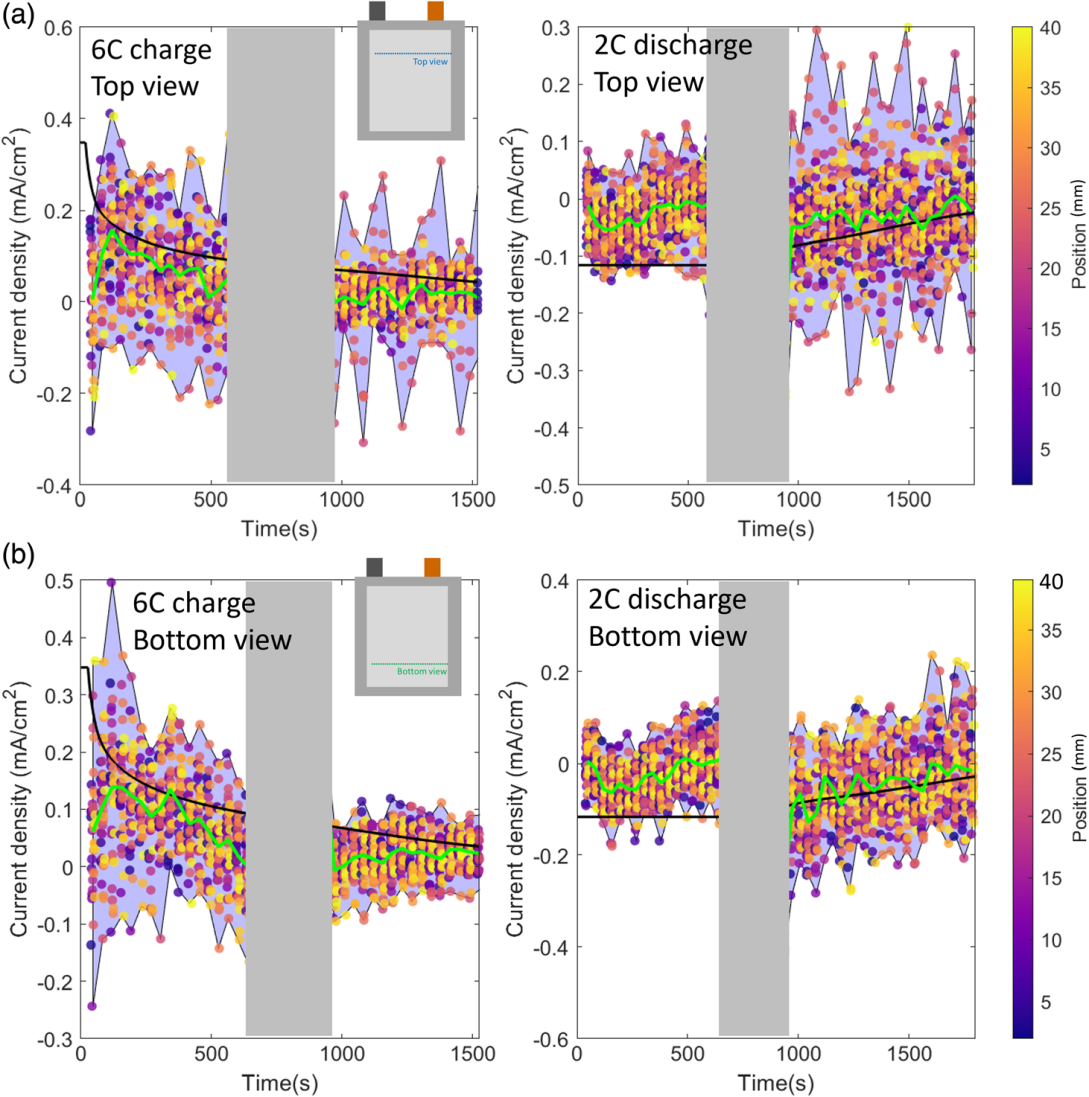
Plots showing the local current density (mA cm^−2^ of graphite surface area) estimated for each time alongside a moving average of current density from the XRD data (green) and the current density estimated using the potentiostat data (black) at the a) top view a) and b) bottom view. The purple semi‐transparent regions captures the max and min bounds. The grayed regions are where disruptions in the measurement occurred and the data were not reliable.

The plots in **Figure** **4** show the distributions of current densities spatially, as well as the averaged current density estimated from the XRD data and the current density estimated from the potentiostat data. Like in previous work,^[^ ^11^ ^]^ the estimates from the potentiostat data are consistently higher than the average current density estimated from the XRD data. The potentiostat recorded electrical data from all phenomena (lithiation/delithiation of graphite, side reactions, and Li plating), whereas the XRD data only quantified the lithiation/delithiation of graphite. Hence, the average from the XRD data should always be lower than what is predicted from the potentiostat. However, while the average current density from the XRD data is lower than that from the potentiostat, some local regions exhibited a higher current density, sometimes by more than 0.1 mA cm^−2^ in the top view and 0.3 mA cm^−2^ in the bottom view. The range of current densities was generally larger for the top view than the bottom view, where the maximum and minimum current densities were consistently higher or lower, respectively, than their counterparts in the bottom view. This was most likely caused by the low‐activity zone that forced relatively high currents in neighboring regions to meet the demand. The average current density along the top view also deviated further away from the potentiostat estimate than the bottom view, indicating that the top region, on average, was less active despite some regions reaching higher current densities.

During discharge, the current density estimated from the XRD data was substantially lower than that estimated from the potentiostat data during the constant current portion of the discharge. This pheneomena were also observed in previous work^[^ ^11^ ^]^ and were attributed to current being initially drawn from Li plating rather than delithiation of graphite.

**Figure 5:**
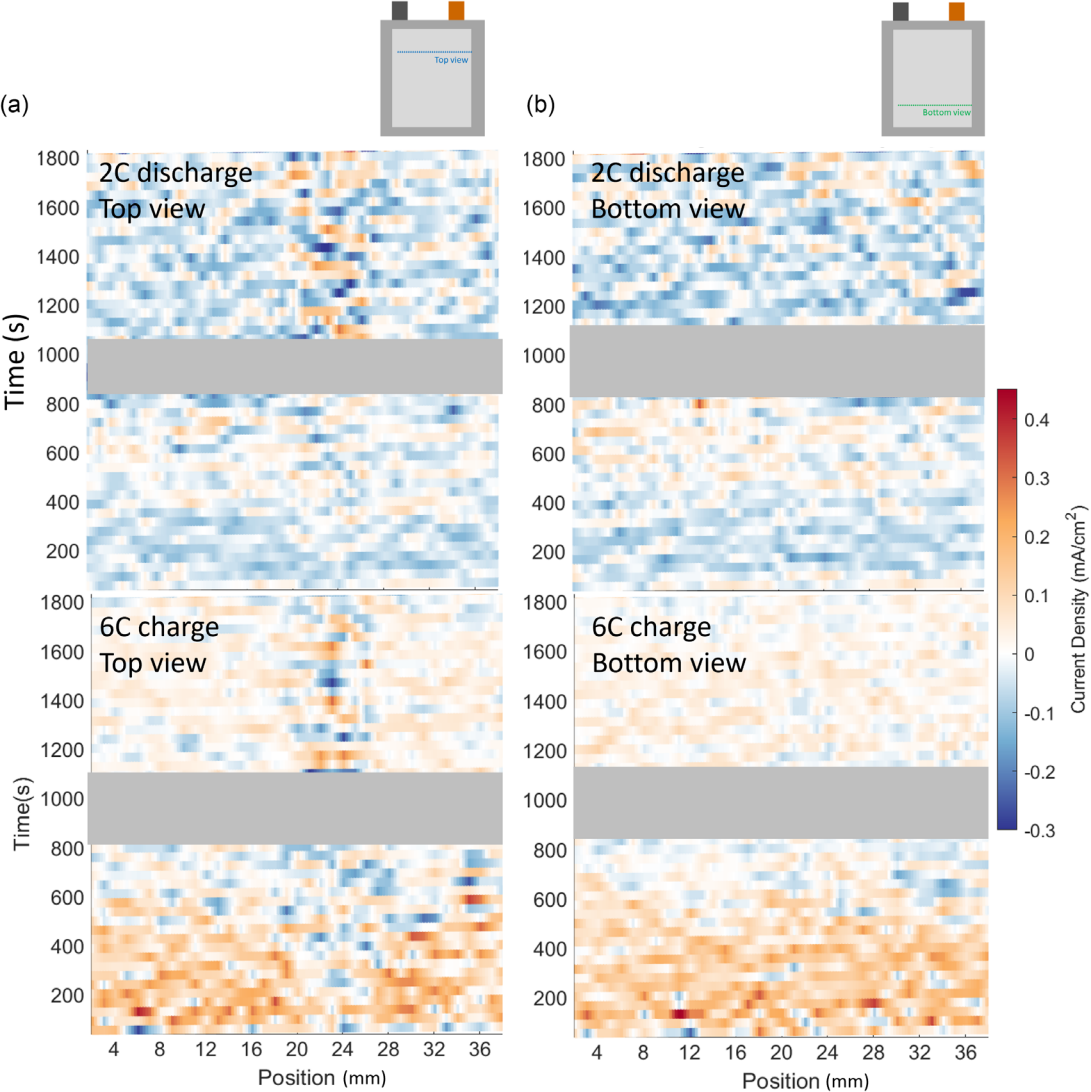
Color maps of current density within the cell at a) the top view and b) the bottom view. The grayed regions are where disruptions in the measurement occurred and the data were not reliable.

Some regions within the cell were observed to have an inverted response during operation, i.e., delithiating during charge and lithiating during discharge. To clarify the extent of positive and negative current locally within the cell, current density maps were plotted representing local lithiation and delithiation, respectively, during charge and discharge, as shown in **Figure** **5**. Figure 5 shows that at any point in time some regions of graphite were undergoing the reverse process of the majority of the material, e.g., during charge, some regions of graphite were delithiating while the majority of graphite within the cell was lithiating. Over the first 400 s of charge and discharge, few regions exhibited reversed activity, but after around 400 s, the amount of reversed activity increased. This time corresponded to when Stage II was mostly present indicating an intermediate stage toward full lithiation or delithiation and perhaps a stage where charge balancing occurred due to neighboring regions having different states of charge and causing local trading of Li ions and electrons within the electrode. Such charge balancing and forward‐back lithiation/delithiation during operation were also observed as a function of depth in previous work.^[^ ^11^ ^]^

### 3.5 Comparing In‐Plane to Through‐Plane Current Density Measurements

**Figure 6:**
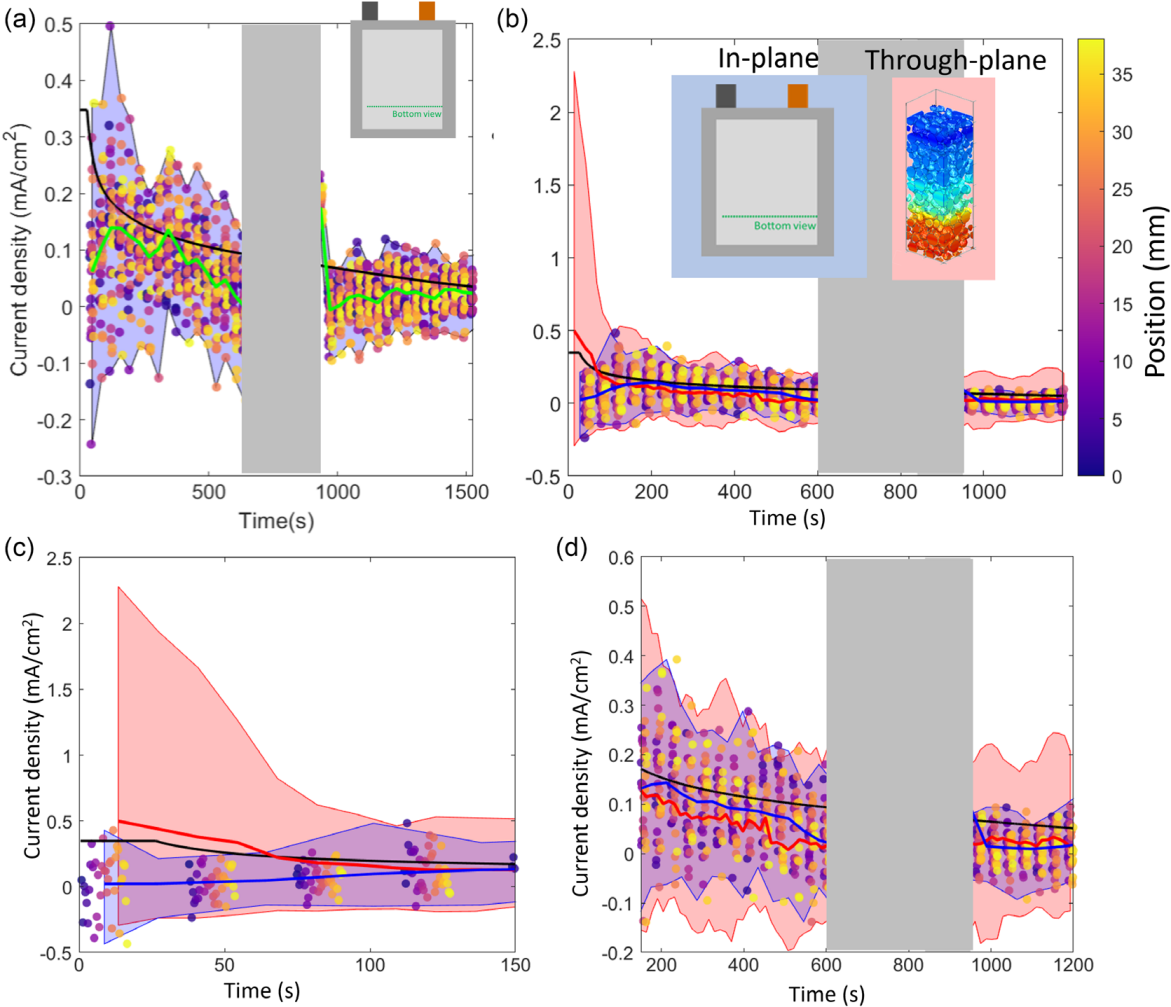
a) Measured current density for in‐plane positions during 6 C charging from XRD data with a moving average plotted in green alongside the average current density estimated from the potentiostat in black. b) The maximum and minimum current densities bound in pink, measured as a function of depth during similar operating conditions of a cell with identical composition in previous work.^[^ ^11^ ^]^ c,d) Magnified regions showing the range of current densities observed in‐plane here (blue) and in previous work as a function of depth (pink). The current at 150 s corresponds to around 3 C. The max and min points of the pink and blue envelopes in (c) are calculated from a moving average of five points, therefore the first five data‐points lie outside the envelope as the first moving average values are acquired. It should be noted that the through‐plane data were collected in a coin cell, while the in‐plane data in this work was collected in a pouch cell. The difference in cell type may cause the behavior to vary.

The XRD point measurements here contain an averaged signal from all depths into the electrode where depth‐resolved composition information cannot be measured. During high‐rate operating conditions of 6C and 2C, severe lithiation gradients are expected along the electrode depth, from separator to current collector. The same cell materials were used in previous work^[^ ^11^ ^]^ that quantified lithiation heterogeneity and lithium plating as a function of depth into the electrode, which found that Li plating occurred for currents greater than 1 mA cm^−2^ of graphite surface area, and that current densities as high as 1.5 mA cm^−2^ existed near the separator while deeper regions towards the current collector were less than 0.1 mA cm^−2^. To elucidate the extent of heterogeneous current densities observed here compared to that observed in the through‐plane direction previously, the two data sets are plotted together in **Figure** **6**. Figure 6a shows the current densities in this work during the 6 C charge with blue bounds following the maximum and minimum values, while Figure 6b–d shows the range of current densities measured in the through‐plane direction from previous work^[^ ^11^ ^]^ with pink bounds following the maximum and minimum values. Data from this work are shown in blue, and data from previous work are shown in pink. The average current density is similar between the summed through‐plane and in‐plane measurements as shown by the blue and red lines in Figure 6c,d. However, the through‐plane information in Figure 6c shows that for the 6 C constant current times of 0–20 s, some regions of graphite experienced an order of magnitude higher current density than the depth‐averaged measurements from the in‐plane profiling in this work. Furthermore, as shown in Figure 6d, the local negative (delithiation) currents during charging also tend to be more extreme than the in‐plane measurements reveal. Therefore, while the in‐plane measurements in this work show substantial heterogeneities in current density across the pouch cell, they did not capture the even greater heterogeneity as a function of depth for each point measured.

## 4 Heterogeneous Pseudo‐3D Modeling

The XRD measurements and interpretation indicate that graphite lithiation heterogeneity is significant in the in‐plane direction. Additionally, these in‐plane heterogeneities can induce local current densities that are on the same scale as through‐plane heterogeneities at lower C rates, e.g., less than 3 C in Figure 6d. In physical Li‐ion battery models, in‐plane heterogeneities are typically neglected in favor of predicting through‐plane lithiation heterogeneities induced by Li‐ion transport resistances. These pseudo‐2D (P2D) models predict that at reasonably fast rates (e.g., >3 C for thicker electrodes),^[^ ^14^ ^]^ through‐plane Li‐ion transport resistances result in overutilization of particles near the separator and underutilization of particles near the current collector. However, the present study indicates that in‐plane as well as through‐plane heterogeneities play a significant role in graphite utilization. To simulate in‐plane lithiation heterogeneities as well as through‐plane heterogeneities, a pseudo‐3D (P3D) model (2D for in‐plane and through‐plane transport and 1D for radial solid‐phase transport) were developed and compared to measured data. By comparing these model results to measurements, the influence/importance of other heterogeneous sources can be evaluated.

### 4.1 Model Results

**Figure 7:**
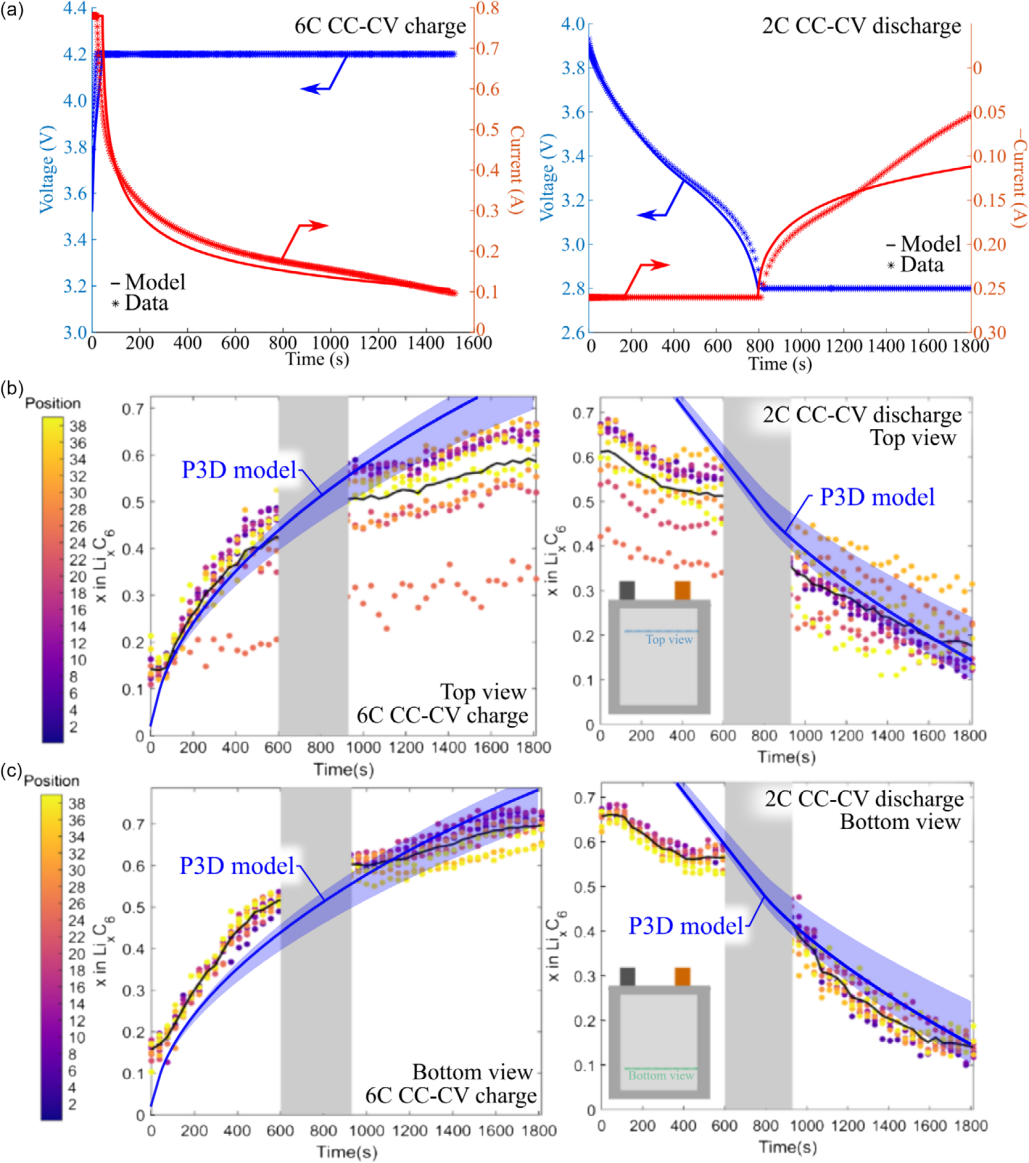
a) Simulated and measured voltage and current during 6 C CC–CV charge (left) and 2 C CC–CV discharge (right). Lithiation state *x* in Li_*x*_C_6_ measured during 6 C charge (left) and 2 C discharge (right) with overlayed modeled distribution from the P3D model for line scans at the b) top view and c) bottom view.

**Figure** **7** compares P3D model simulation results and experimental data. Figure 7a illustrates model validation of the 6 C CC–CV charge (left) and 2 C CC–CV discharge (right). As illustrated, the model captured both the voltage and current response well throughout the cycle. There was a slight discrepancy in current during the 2.8 V hold during the discharge. Here, the model predicted less cell‐level resistance than measured experimentally. However, the overall model prediction is quite good. Figure 7b,c illustrates the averaged in‐plane lithiation state predicted from the model overlayed on data taken at the top view (Figure 7b) and bottom view (Figure 7c) during charge and discharge. During charge, the model‐predicted lithiation variance was smaller than measured experimentally. In particular, the top‐view XRD scan measured sections that were severely underutilized. This underutilized region may have been a result of Li‐plating shadow effects.^[^ ^31^ ^]^ Additionally, near the end of charge where plating was most likely present (and not modeled), the model predicted continual increase in graphite intercalation fractions, while the experiment showed significantly less graphite utilization. This slope discrepancy was a strong indicator of Li being plated as opposed to being intercalated into the graphite. During discharge, the model and experiment disagree during the initial 600 s (a significant portion of the CC section). The model predicts that the 2 C rate did not induce significant lithiation gradients, and thus the 2 C discharge current was demanded uniformly. Conversely, the measured data indicate very slight changes in lithiation during this CC discharge portion. This indicates that either 1) other sections of the cell contributed significantly to compensate for the measured decreased utilization or more likely, 2) Li‐stripping reactions contributed significantly to the current in this CC portion. Li plating and stripping were not simulated, and thus in the simulation, the current came from delithiating the graphite. This mismatch in current sources resulted in poor model‐experiment overlap at the beginning of discharge. Despite this discrepancy, the averaged model‐predicted range of through‐plane lithiation heterogeneities encompassed a majority of the measured lithiation ranges.

**Figure 8:**
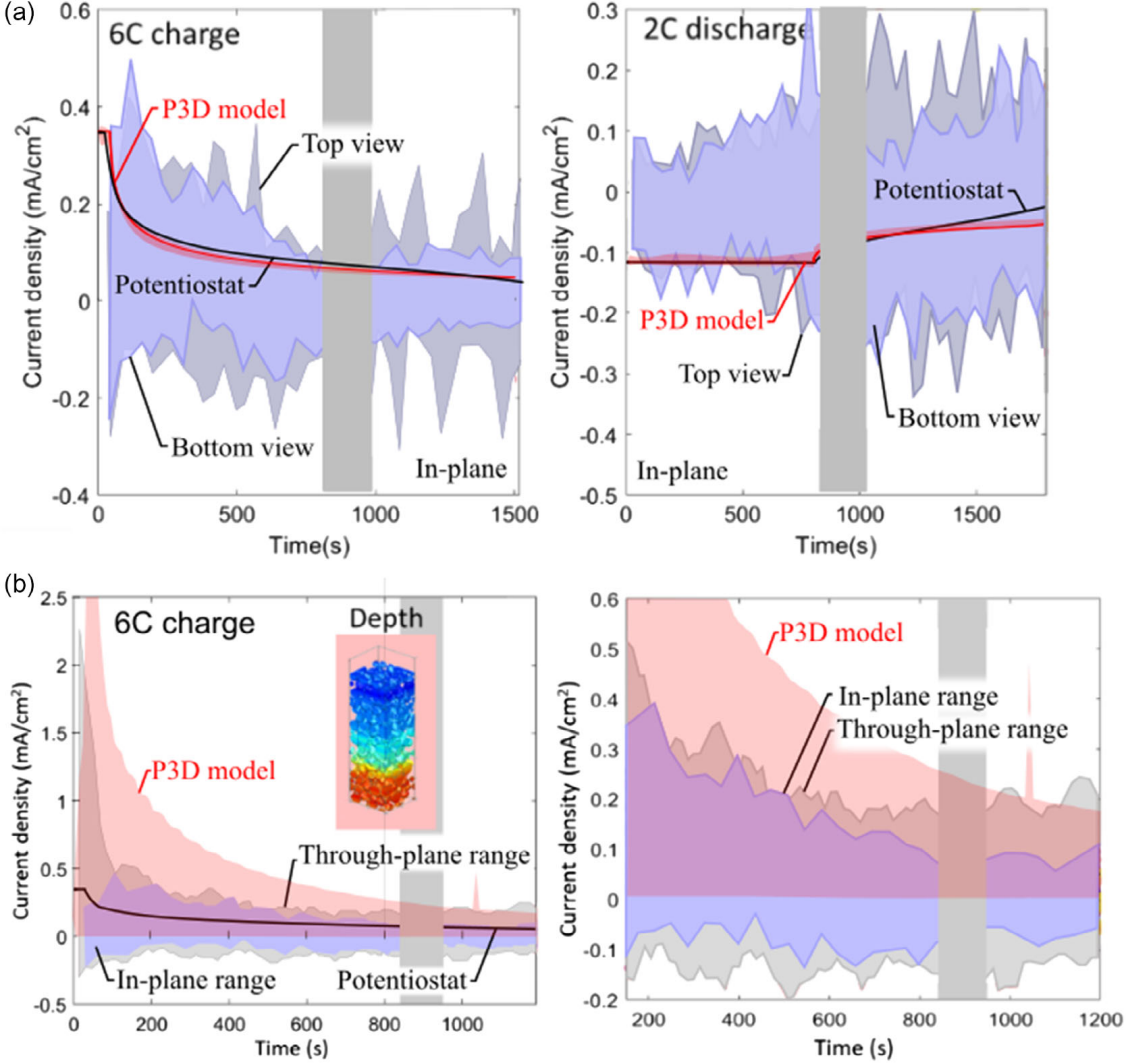
a) Local in‐plane current density estimated for the top and bottom view from the XRD experiment alongside the current density estimated from the potentiostat (black) and the model‐predicted ranges (red) under 6 C charge (left) and 2 C discharge (right). b) Through‐plane current density ranges under 6 C charge. Illustrated are the predicted through‐plane ranges (red), the through‐plane ranges reported in previous work^[^ ^11^ ^]^ (gray), and the in‐plane measurements from the bottom view (blue).

The model‐predicted range of through‐plane lithiation heterogeneities encompassed a majority of the measured lithiation ranges (Figure 7). However, the model did not capture the range in local current densities. **Figure** **8**a illustrates the in‐plane local current densities predicted by the model and measured experimentally. The local current density is measured to have regions with both positive and negative values. This means that during charging, there are certain regions of graphite lithiating, while other sections are delithiating. As illustrated, the modeled range (red region) is miniscule compared to the range measured experimentally and is always the sign of the potentiostat (i.e., no regions are lithiating while others are delithiating). The model‐experiment discrepancy is probably due to the staging dynamics of graphite and Li‐plating dynamics, which are not captured in standard pseudo‐2D models and require a higher level of theory and/or knowledge of grain orientation/nucleation sites. Importantly, heterogeneous electrolyte transport alone cannot explain the large in‐plane heterogenous current densities extracted from XRD.

Figure 8b illustrates 1) the through‐plane current density range measured in a previous study,^[^ ^11^ ^]^ 2) the through‐plane heterogeneities predicted by the P3D model, and 3) the in‐plane current density measured at the bottom view. All results are taken during 6 C CC–CV charging. As illustrated, the P3D model predicted significantly more heterogeneity in the through‐plane direction as compared to the in‐plane direction (Figure 7). Additionally, the model‐predicted variance is above or near that of the measured through‐plane response. The model also predicted that during charge, almost all the graphite is lithiating, while the data show significant delithiation can occur during charging (including during the fast 6 C portion). There are instances where the model predicted delithiation during charging. For instance, at the beginning of charge, the particles near the separator are highly utilized, while particles near the current collector are highly underutilized. During the CV portion, the model can predict that the particles near the separator begin to delithiate to help charge the particles near the current collector. However, for this cell build and parameters used in the present model, such balancing was not seen significantly. An important observation here is that physics‐based models did predict through‐plane heterogeneous local current densities on the same range or above those measured experimentally (Figure 8b). This means that for Li‐plating considerations, the computed through‐plane current densities are conservative. However, the model underpredicted the in‐plane heterogeneities (Figure 8a), which may need to be considered when designing Li‐plating conscious charging protocols.

## 5 Conclusions

High‐speed synchrotron XRD taken in‐plane across the face of a pouch cell during 6 C fast charge and 2 C discharge revealed that the graphite anode exhibited considerable spatial variation in lithiation and delithiation response during operation. During charge, following constant current constant voltage, it was shown that some regions within the cell quickly reached LiC_6_, while other regions never fully lithiated to LiC_6_. Consequences of this would likely include charge balancing over millimeters distance when the cell is left at open circuit for some time. During discharge, it was also shown that while most of the negative electrode transitioned from LiC_6_ to graphite, some regions still contained LiC_6_ even at full discharge to 2.8 V, indicating that some graphite became unresponsive to discharge operating conditions which may have been due to Li plating.

The time‐resolved XRD data were used to estimate the local current density as mA cm^−2^ of graphite surface area. While the average current density followed the expected trend during charge and discharge, local current densities displayed a vast range of values that included short‐lived and localized inverted currents, i.e., some regions displayed discharge behavior while the cell was charging, and vice versa. Consequently, while some regions displayed inverted behavior, other regions displayed larger current densities than the mean calculated using the potentiostat current. A P3D model was developed to estimate the variation in current density in the in‐plane and through‐plane directions, but while the model was able to conservatively predict current density gradients through‐plane, it greatly underestimated the variation in in‐plane current density, even upon varying local transport parameters like electrode tortuosity.

This extreme in‐plane heterogeneity in current density may have been due to the local phase transitioning and phase balancing between particles which is not yet well understood, nor easily modelled across large areas of porous electrode without advanced computing capabilities. However, it is expected that such heterogeneous behavior will have significant consequences for physics‐based charging protocols that set current limits under the assumption of homogeneous in‐plane electrochemical response. Some regions have a higher propensity to plate than others, which may help explain the often highly localized occurrence of Li‐plating within cells following fast charging. In‐plane lithiation heterogeneities are still not well understood, nor are solutions to reducing the in‐plane heterogeneity being widely explored. Yet, as this work shows, investigating in‐plane heterogeneity is an important direction for research to pursue to achieve controlled, well‐predicted cycle behaviors to utilize cells to their full potential.

## Conflict of Interest

The authors declare no conflict of interest.

## Supporting information

Supplementary Material

## Data Availability

The data that support the findings of this study are available from the corresponding author upon reasonable request.
