# Supplementary material for: Dynamic In‐Plane Heterogeneous and Inverted Response of Graphite to Fast Charging and Discharging Conditions in Lithium‐Ion Pouch Cells
Source: Small Sci. 2023 Apr 19;3(7):2200067. doi: 10.1002/smsc.202200067 (PMC11935889; doi:10.1002/smsc.202200067)
Supplement: Supplementary file 1 — Supplementary Material [file SMSC-3-2200067-s001.pdf]

Supplementary Information

Dynamic in-plane heterogeneous response of graphite to fast charging and discharging conditions in pouch cells

Abigale Monasterial[1#], Peter J. Weddle[1#], Kristen Atkinson[1], David Wragg[2], Andrew Colclasure[1], Francois Usseglio-Viretta[1], Natalie Seitzman[3], Jun-Sang Park[4], Jonathan Almer[4], Kandler Smith[1], Donal Finegan[1]*

1. National Renewable Energy Laboratory, 15013 Denver West Parkway, Golden, Colorado 80401, United States
2. Centre for Materials and Nanotechnology, University of Oslo, 0315 Oslo, Norway
3. Colorado School of Mines, 1500 Illinois St, Golden, Colorado 80401, United States
4. X-ray Science Division, Advanced Photon Source, Argonne National Laboratory, Lemont, Illinois 60439, United States

# Equally contributing first authors

*Corresponding author: donal.finegan@nrel.gov

1. Information on fitting X-ray diffraction data

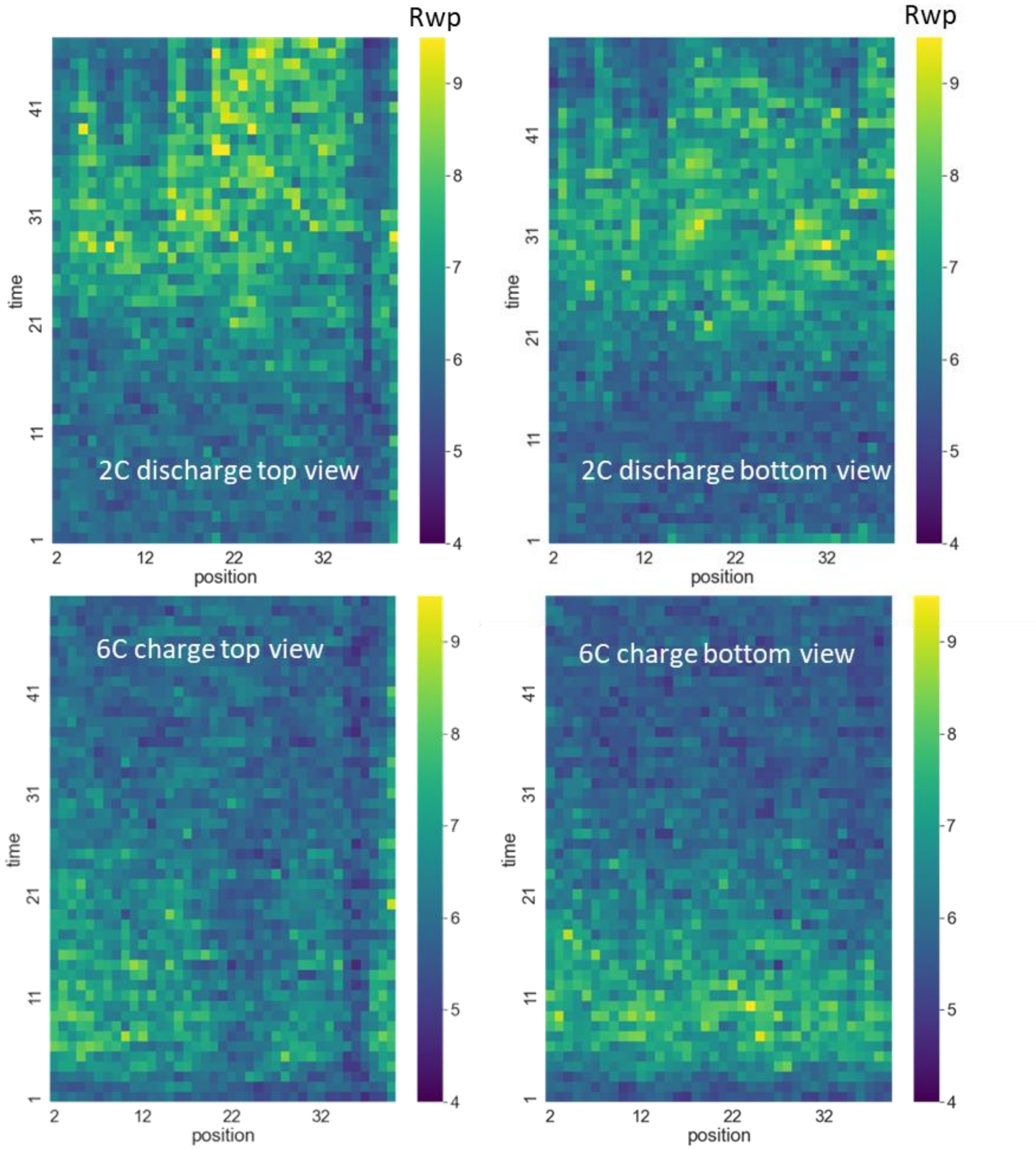

Figure S1. Rwp maps in time and space for the Rietveld fits used to produce the data in figures 2 and 3 of the main manuscript. The missing experimental time period is not shown in the plots. Time and position in arbitrary units.

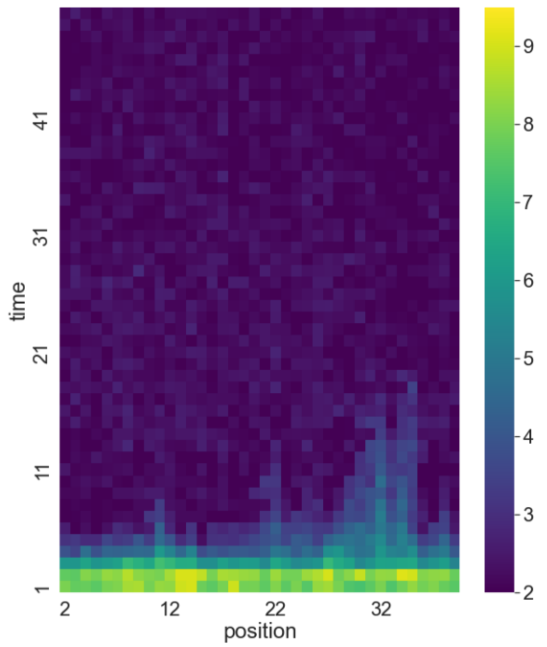

Graphite: model without stage 4, full pattern fit

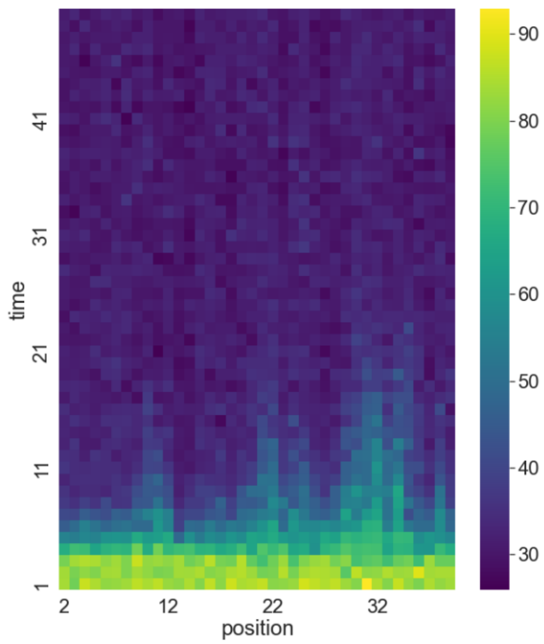

Graphite: model without stage 4, fit limited to graphite peak region

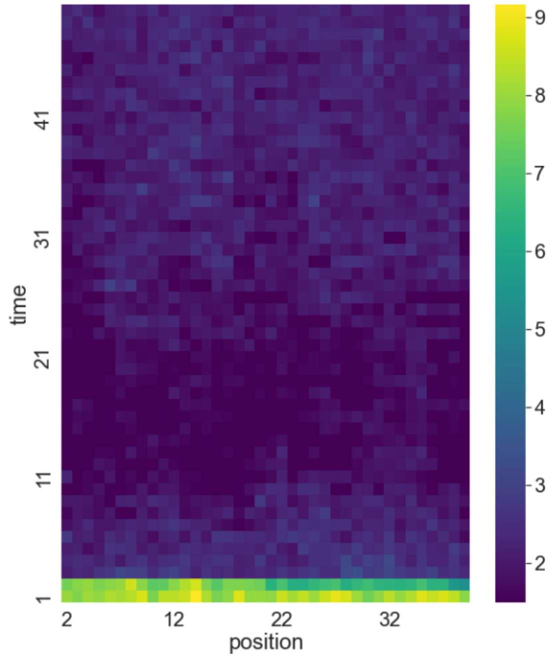

Graphite: model including stage 4.

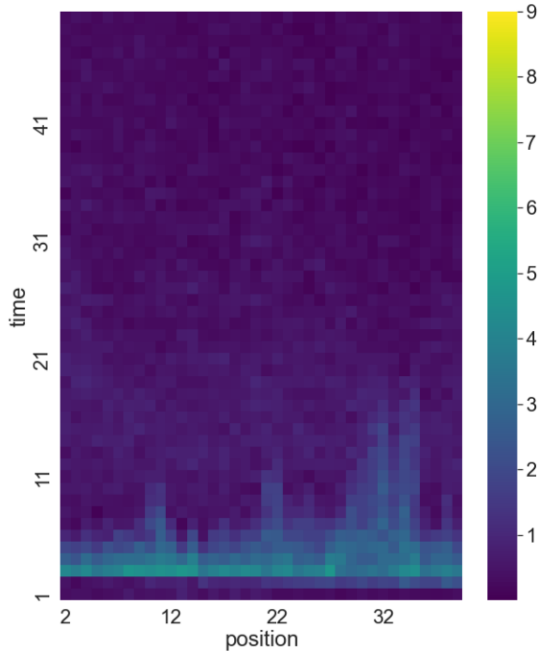

Stage 4: model including stage 4.

Figure S2. Graphite and stage 4 weight % distributions in time and space (6C charge, bottom view) using various fitting models. The same time/space distribution is observed (1, top row) independent of whether the full pattern or just the graphite peak region is fitted; (2, top left vs bottom row) for the sum of the graphite and stage 4 distributions compared to graphite in a model where stage 4 is not fitted.

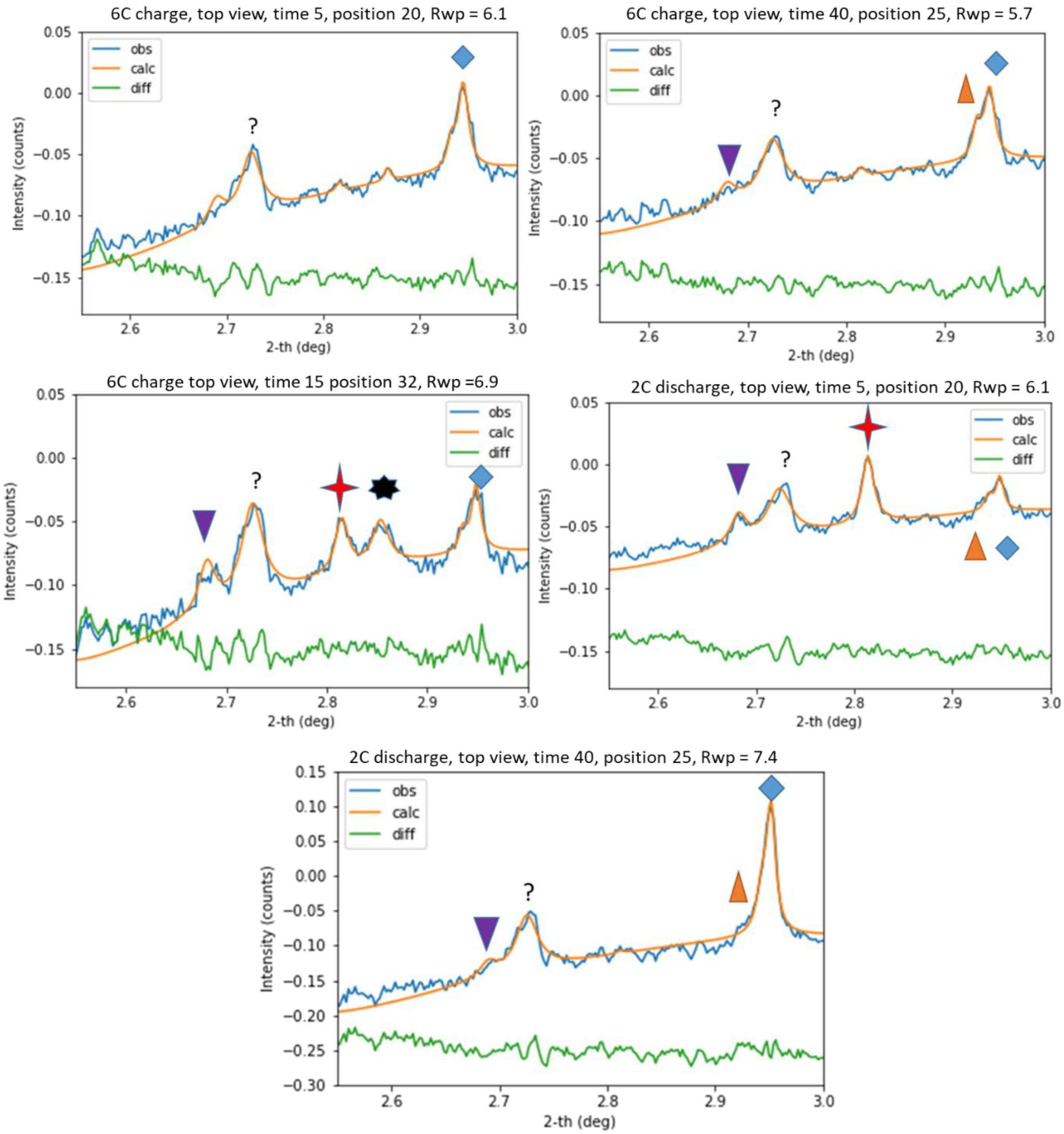

Figure S3. Rietveld fits at selected positions in the top view and times in the dataset. The blue lines represent the observed data, orange lines are the fits, and the green line is the difference. The three unknown material peaks are marked with question marks. For each diffraction pattern the full fitted range is shown first with a zoom-in on the range where the LiCx peaks are observed below. The LiCx peaks are marked in the zoomed plots: blue diamond = graphite, orange wedge = stage 4, black star =LiC30, red star = LiC12, and the purple wedge = LiC6.

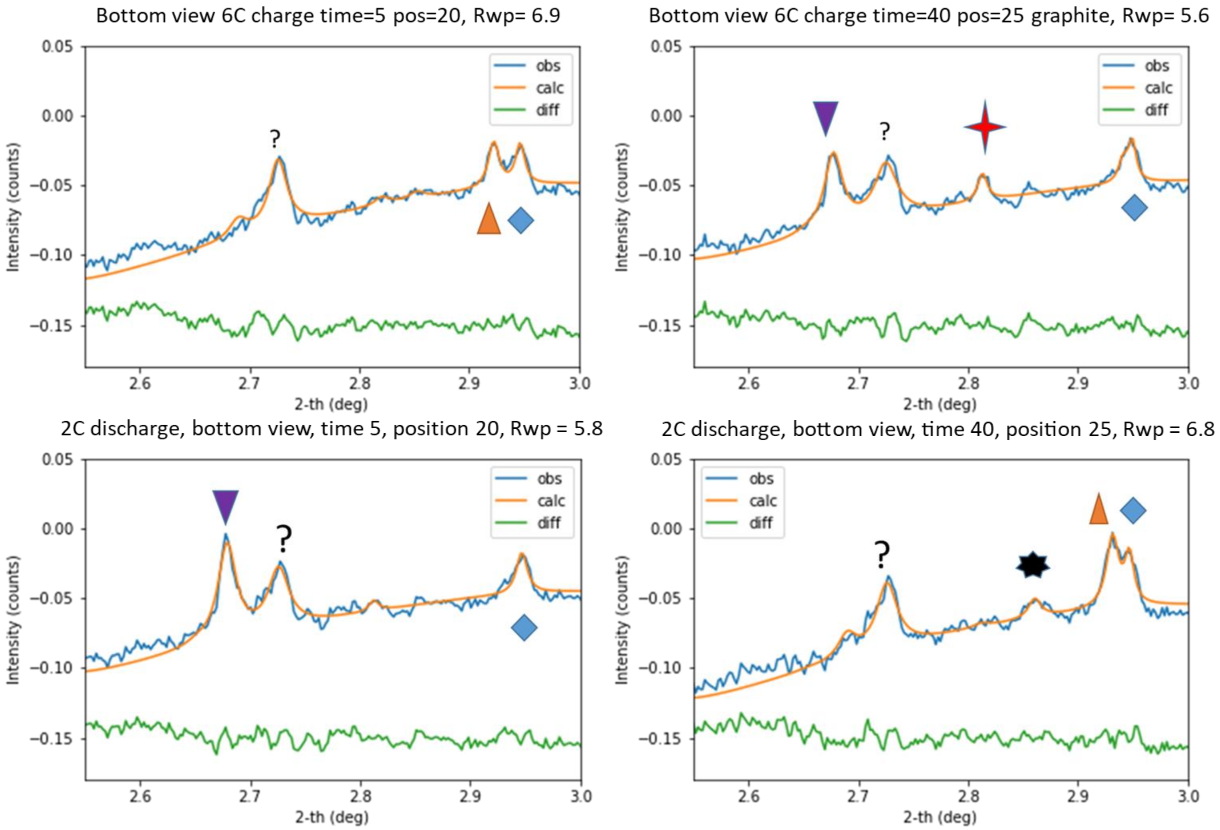

Figure S4. Rietveld fits at selected positions in the bottom view and times in the dataset. The blue lines represent the observed data, orange lines are the fits, and the green line is the difference. The three unknown material peaks are marked with question marks. For each diffraction pattern the full fitted range is shown first with a zoom-in on the range where the LiCx peaks are observed below. The LiCx peaks are marked in the zoomed plots: blue diamond = graphite, orange wedge = stage 4, black star =LiC30, red star = LiC12, and purple wedge = LiC6.

Example TOPAS input file:

```
conserve_memory
approximate_A
	A_matrix_memory_allowed_in_Mbytes 100
bootstrap_errors 200
'time 1 position 2
r_exp  412.716476 r_exp_dash  725.936809 r_wp  6.15608152 r_wp_dash  10.828078 r_p -6.13603718
r_p_dash  26.7576736 weighted_Durbin_Watson  0.301989586 gof  0.0149160062
iters 100000

'diffraction data file location
xdd "M:\2020_nrel_linescans\pcell1\pcell1_4\pcell1_4_1_2.xy"
	r_exp  412.716476 r_exp_dash  725.936809 r_wp  6.15608152 r_wp_dash  10.828078 r_p -6.13603718
r_p_dash  26.7576736 weighted_Durbin_Watson  0.301989586 gof  0.0149160062
	x_calculation_step 0.002
	bkg @ -0.213377606`_0.000743422633  0.156849688`_0.0011599091  3.0896971e-
005`_0.00112515418 -0.018348727`_0.00128842808 -0.0157075483`_0.0010752388
0.00419231177`_0.00101559645
	start_X  1.5
	finish_X  3.2
	LP_Factor( 90)' synchrotron radiation
'wavelength and emmission profile
	lam
		ymin_on_ymax  0.001
		la  1 lo  0.1729786 lh  0.00501844
'structural information
	str
	local !a_graphite 2.384782653_LIMIT_MIN_2.35 min 2.35 max 2.4
	local c_graphite  6.73047`_0.00307 min 6.7 max 6.8
		'LVol_FWHM_CS_G_L( 1, 39.21458_4.43906, 0.89, 54.82232_6.20584,,,cs_1200017,
61.59811_6.97285 min =30;)
		r_bragg  100
		phase_MAC  0.165838073
		phase_name "Graphite 2H"
		MVW( 48.043, 33.149`_0.015, 9.011_0.683)
		space_group P_63_m_c
		scale sc_1200017  1.10839477e-009`_2.953e-010
		Phase_LAC_1_on_cm( 0.39911`_0.00018)
		Phase_Density_g_on_cm3( 2.40660`_0.00110)
		'Hexagonal(!a_1200017 2.384782653_LIMIT_MIN_2.35 min =2.35; max =2.4;,c_1200017
6.742433_0.002820 min =6.7; max =6.8;)
		'a !a_1200017 2.384782653_LIMIT_MIN_2.35 min =2.35; max =2.4;
		a = a_graphite; :  2.384783
		b = Get(a);
		c =c_graphite; :  6.730467`_0.003070
		'c c_1200017  6.721680_0.000908 min =6.7; max =6.8;
		ga 120
		site c_1 num_posns  2 x =0; :  0.00000 y =0; :  0.00000 z !z_c_1_1200017 0 occ c
!occ_c_1_1200017 1 beq !beq_c_1_1200017 1
		site c_2 num_posns  2 x =1/3; :  0.33333 y =2/3; :  0.66667 z !z_c_2_1200017 0.005 occ c
!occ_c_2_1200017 1 beq !beq_c_2_1200017 1
```

```
			CS_L(csl_graphite_1_2, 75.63452`_29.85724_LIMIT_MIN_50 min 50 max 150)
			weight_percent  2.365`_0.615
'output of parameters to file
			out "Results_graphite_region.txt" append
				Out(Get(r_wp), "Rwp; 1_2; %11.5f ;")
				Out(Get (weight_percent), "percent_graphite 2H; %11.5f ;", " %11.5f ;")
				'Out(a_graphite, " a_graphite; %11.5f ;", " %11.5f ;")
				Out(c_graphite, " c_graphite; %11.5f ;", " %11.5f ;")
				Out(csl_graphite_1_2,  " CSL graphite; %11.5f ;", " %11.5f ;")
		str
		/*
		_cell_length_a 2.46657(23)
_cell_length_b 2.46657(23)
_cell_length_c 20.8117(4)
		*/
		local c_li033c		20.83226`_749.85248_LIMIT_MIN_20.6 min =20.6; max =21.0;
		'LVol_FWHM_CS_G_L( 1, 190.90878_1616.94835, 0.89, 266.89213_2260.50790,,,@,
299.87880_2539.89652_LIMIT_MIN_150 min =150; max =300;)
			r_bragg  17210312.6
			phase_MAC  0.165326543
			phase_name "Li0-033C"
			MVW( 146.905, 109.762`_3950.876, 0.000_0.487)
			space_group P63/mmc
			scale @  1.00002174e-015`_2.473e-011_LIMIT_MIN_1e-015
			Phase_LAC_1_on_cm( 0.36743`_13.22554)
			Phase_Density_g_on_cm3( 2.22245`_79.99646)
			'Hexagonal( 2.46657,@  20.798656_0.040577 min =20.5; max =21.0;)
			a 2.46657
			b = Get(a);
			c = c_li033c; :  20.832258`_749.852476
			ga 120
			site C1 num_posns  2 x  0 y  0 z  0.25 occ C  1 beq  0.025
			site C2 num_posns  2 x =1/3; :  0.33333 y =2/3; :  0.66667 z  0.25 occ C  1 beq  0.025
			site C3 num_posns  4 x =1/3; :  0.33333 y =2/3; :  0.66667 z  0.4135 occ C  1 beq  0.025
			site C4 num_posns  4 x =2/3; :  0.66667 y =1/3; :  0.33333 z  0.4135 occ C  1 beq  0.025
			site Li1 num_posns  2 x  0 y  0 z  0 occ Li  0.2 beq  0.03
			CS_L(csl_li033c_1_2, 98.38710`_3892745.98295_LIMIT_MIN_50 min 50 max 150)
			weight_percent  0.000`_0.512
			out "Results_graphite_region.txt" append
				Out(Get (weight_percent), "percent_Li0-033C; %11.5f ;", " %11.5f ;")
				Out(c_li033c, " c_li0-033c; %11.5f ;", " %11.5f ;")
				Out(csl_li033c_1_2,  " CSL li0-033c; %11.5f ;", " %11.5f ;")
		str
		/*
		_cell_length_a 4.29311(12)
_cell_length_b 4.29311(12)
_cell_length_c 7.0358(5)
*/
		local	c_lic12  7.04248`_0.00303 min =6.9; max =7.1;
		'LVol_FWHM_CS_G_L( 1, 127.32395_0.00490, 0.89, 178.00000_0.00684,,,@,
200.00000_0.00769_LIMIT_MIN_50 min =50; max =300;)
			r_bragg  10853054.7
			phase_MAC  0.164594407
```

```
			phase_name "LiC12"
			MVW( 151.058, 112.409`_0.048, 0.000_0.000)
			space_group P6/mmm
			scale @  1.69770908e-010`_3.316e-011
			Phase_LAC_1_on_cm( 0.36729`_0.00016)
			Phase_Density_g_on_cm3( 2.23148`_0.00096)
			'Hexagonal( 4.29311,@  7.027546_0.021895_LIMIT_MIN_7 min =7; max =7.1;)
			a 4.29311
			b = Get(a);
			c = c_lic12;:  7.042483`_0.003026
			ga 120
			site Li1 num_posns  1 x  0 y  0 z  0 occ Li  1 beq  0.0038
			site C1 num_posns  12 x =1/3; :  0.33333 y  0 z  0.2352 occ C  0.99992 beq  0.0038
			CS_L(csl_lic12_1_2, 61.95001`_17.68533_LIMIT_MIN_50 min 50 max 150)
			weight_percent  3.863`_0.712
			out "Results_graphite_region.txt" append
				Out(Get (weight_percent), "percent_LiC12; %11.5f ;", " %11.5f ;")
				Out(c_lic12, " c_lic12; %11.5f ;", " %11.5f ;")
				Out(csl_lic12_1_2,  " CSL lic12; %11.5f ;", " %11.5f ;")

	str
	local c_lic6		3.70468`_0.00209 min =3.685; max =3.715;
	/* CIF values
	_cell_length_a 4.31613(5)
_cell_length_b 4.31613(5)
_cell_length_c 3.69986(6)
*/
	'LVol_FWHM_CS_G_L( 1, 19.09859_6.14840, 0.89, 26.70000_8.59552,,,@,
30.00000_9.65789_LIMIT_MIN_30 min =30; max =300;)
			r_bragg  43983.7508
			phase_MAC  0.163460012
			phase_name "LiC6"
			MVW( 78.999, 59.768`_0.034, 3.028_0.840)
			space_group P6/mmm
			scale @  7.07890655e-010`_1.405e-010
			Phase_LAC_1_on_cm( 0.35877`_0.00020)
			Phase_Density_g_on_cm3( 2.19484`_0.00124)
			'Hexagonal( 4.31613,@  3.650000_0.004442_LIMIT_MIN_3.65 min =3.65; max =3.73;)
			a 4.31613
			b = Get(a);
			c = c_lic6; :  3.704685`_0.002087
			ga 120
			site Li1 num_posns  1 x  0 y  0 z  0 occ Li  1 beq  0.0038
			site C1 num_posns  6 x  0 y  0.3257 z  0.5 occ C  0.99992 beq  0.0038
			CS_L(csl_lic6_1_2, 50.00000`_13.90567_LIMIT_MIN_50 min 50 max 150)
			weight_percent  4.479`_0.846

			out "Results_graphite_region.txt" append
				Out(Get (weight_percent), "percent_LiC6; %11.5f ;", " %11.5f ;")
				Out(c_lic6, " c_lic6; %11.5f ;", " %11.5f ;")
				Out(csl_lic6_1_2,  " CSL lic6; %11.5f ;", " %11.5f ;\n")

'LMNO side
```

```
str
local c_lmno1  14.35102`_0.00106 min =14.2; max =14.5;
	'LVol_FWHM_CS_G_L( 1, 47.95169_33770865.47711, 0.89, 49.84590_27659152.05720,@,
63.65805_28771719.18194 min =50;,@, 261.48906_785329744.14618 min =50;)
	r_bragg  142.65604
	phase_MAC  0.556485929
	phase_name "LMNO1"
	MVW( 285.790, 163.781`_0.012, 85.049_1.331)
	space_group R-3mH
	scale @  4.1360839e-010`_5.682e-012
	Phase_LAC_1_on_cm( 1.61245`_0.00012)
	Phase_Density_g_on_cm3( 2.89756`_0.00021)
	'Trigonal(@  3.632114_0.005775,@  14.258447_0.000179 min =14.2; max =14.5;)
	a   3.630153_0.006609
	b = Get(a);
	c = c_lmno1;
	ga 120
	site Li1 num_posns  3 x  0 y  0 z  0.5 occ Li+1  0.94 beq  0
	site Ni1 num_posns  3 x  0 y  0 z  0.5 occ Ni+2  0.038 beq  0
	site Ni2 num_posns  3 x  0 y  0 z  0 occ Ni+2  0.44 beq  0
	site Mn1 num_posns  3 x  0 y  0 z  0 occ Mn+4  0.3 beq  0
	site Co1 num_posns  3 x  0 y  0 z  0 occ Co+3  0.2 beq  0
	site Li2 num_posns  3 x  0 y  0 z  0 occ Li+1  0.06 beq  0
	site O1 num_posns  6 x  0 y  0 z  0.26 occ O-2  1 beq  0
	weight_percent  25.943`_0.479
	CS_L(csl_lnmo_1_2, 155.47224`_12.60621 min 50 max 400)
	CS_G(csg_lnmo_1_2, 73.26872`_1.60391 min 50 max 400)

		out "Results_LMNO_region.txt" append
		Out(Get (weight_percent), "percent_LMNO1; %11.5f ;", " %11.5f ;")
		Out(c_lmno1, " c_lmno1; %11.5f ;", " %11.5f ;")
		Out(csl_lnmo_1_2,  " CSL LMNO; %11.5f ;", " %11.5f ;")
		Out(csg_lnmo_1_2,  " CSG LMNO; %11.5f ;", " %11.5f ;")
str
local c_lmno2       14.49345`_0.00043 min =14.3; max =15;
'LVol_FWHM_CS_G_L( 0.89, 63.50549_17360559.83749, 0.89, 67.59950_18479743.38518,@,
75.95449_20763756.61411 min =50;,@, 1737879306000.00000_0.01664 min =50;)
	r_bragg  123.976109
	phase_MAC  0.556485929
	phase_name "LMNO2"
	MVW( 285.790, 165.406`_0.005, 1.966_0.383)
	space_group R-3mH
	scale @  1.000081e-009`_1.183e-011
	Phase_LAC_1_on_cm( 1.59661`_0.00005)
	Phase_Density_g_on_cm3( 2.86909`_0.00008)
	'Trigonal(@  3.601771_1.290873,@  14.496003_0.021570 min =14.3; max =15;)
	'a   3.683669_0.006619
	a 3.630153
	b = Get(a);
	c = c_lmno2; :  14.493447`_0.000428
	ga 120
	site Li1 num_posns  3 x  0 y  0 z  0.5 occ Li+1  0.94 beq  0
	site Ni1 num_posns  3 x  0 y  0 z  0.5 occ Ni+2  0.038 beq  0
```

```
			site Ni2 num_posns  3 x  0 y  0 z  0 occ Ni+2  0.44 beq  0
			site Mn1 num_posns  3 x  0 y  0 z  0 occ Mn+4  0.3 beq  0
			site Co1 num_posns  3 x  0 y  0 z  0 occ Co+3  0.2 beq  0
			site Li2 num_posns  3 x  0 y  0 z  0 occ Li+1  0.06 beq  0
			site O1 num_posns  6 x  0 y  0 z  0.26 occ O-2  1 beq  0
			weight_percent  63.350`_0.954
			CS_L(csl_lnmo_1_2, 155.47224`_12.60621 min 50 max 400)
			CS_G(csg_lnmo_1_2, 73.26872`_1.60391 min 50 max 400)

			out "Results_LMNO_region.txt" append
				Out(Get (weight_percent), "percent_LMNO12; %11.5f ;", " %11.5f ;")
				Out(c_lmno2, " c_lmno2; %11.5f ;", " %11.5f ;\n")

'unknown pks
		xo_Is
		xo xpk1_1_2  2.48905687`_0.000278982098 min 2.45 max 2.5
			peak_type fp
			'LVol_FWHM_CS_G_L( 1, 30.57661_1.44628, 0.89, 42.74636_2.02192,,,@, 48.02962_2.27182)
			I intpk1_1_2  0.00000`_0.00000
			CS_L(csl_pk1_1_2, 47.34960`_2.28620 min 15 max 100)


		xo_Is
			xo xpk2_1_2  2.22622943`_0.000470903846 min 2.15 max 2.3
			peak_type fp
			'LVol_FWHM_CS_G_L( 1, 62.77881_9.72696, 0.89, 87.76532_13.59838,,,@,
98.61272_15.27907)
			I intpk2_1_2  0.00000`_0.00000
			CS_L(csl_pk2_1_2, 92.55334`_15.05745_LIMIT_MAX_120 min 15 max 120)


		xo_Is
			xo xpk3_1_2  2.72542048`_0.00150371647_LIMIT_MIN_2.72 min 2.72 max 2.74
			peak_type fp
			'LVol_FWHM_CS_G_L( 1, 38.19719, 0.89, 53.40000,,,, 60)
			I intpk3_1_2  0.00000`_0.00000
			CS_L(csl_pk3_1_2, 36.28905`_7.42111 min 15 max 100)

out "Results_pks.txt" append
				Out(xpk1_1_2, "pos pk1; %11.5f ;", " %11.5f ;")
				Out(100000*intpk1_1_2, " scaled int pk1; %11.5f ;", " %11.5f ;")
				Out(csl_pk1_1_2, " csl pk1; %11.5f ;", " %11.5f ;")
				Out(xpk2_1_2, "pos pk2; %11.5f ;", " %11.5f ;")
				Out(100000*intpk2_1_2, " scaled int pk2; %11.5f ;", " %11.5f ;")
				Out(csl_pk2_1_2, " csl pk2; %11.5f ;", " %11.5f ;")
				Out(xpk3_1_2, "pos pk3; %11.5f ;", " %11.5f ;")
				Out(100000*intpk3_1_2, " scaled int pk3; %11.5f ;", " %11.5f ;")
				Out(csl_pk3_1_2, " csl pk3; %11.5f ;", " %11.5f ;\n")
'END
```

2. **Estimating lithiation state and current density**

Variable parameters

x	x in Li_xC_6	t	Time (s)
f_i	Mass fraction of lithium in phase i	I	Current (A)
m_i	Mass fraction of phase i	$I_{density}$	Current density (A/g)
MW_i	Molecular weight of phase i	I_{local}	Current at a specific position (A)
M_i	Mass of phase i		

Fixed parameters

$\rho_{gr.areal}$	Areal density of graphite	0.01397 g/cm^2	$A_{sp.gr}$	Specific surface area graphite	0.89 m^2/g
ρ_{gr}	Density of graphite	1.38 g/cm^3	F	Faraday's constant	96485.3 C/mol
h_{gr}	Height of graphite electrode	101 µm			

Calculating x in Li_xC_6 and capacity from XRD data

To calculate the lithiation state x in Li_xC_6, i.e. the number of moles of Li per mole of C_6, from the mass fractions of stages determined from XRD, compositions were assigned to the mass fractions of each phase determined by Rietveld refinement (m_{stageI}, $m_{stageII/IIL}$, $m_{stageIII}$). The stages that were quantified were graphite, Stage III, Stage II, and Stage I. The following compositions were used for each phase:

1. Graphite: C_6
2. Stage III: LiC_{18}
3. Stage II: LiC_{12}
4. Stage I: LiC_6

Using these compositions, the lithiation state x in Li_xC_6 was calculated from the XRD-determined mass fractions using the following equation:

$$x = 6\left(\frac{\frac{f_{StageIII} \times m_{Stage\ III} + f_{StageII} \times m_{StageII} + f_{StageI} \times m_{StageI}}{MW_{Li}}}{\frac{m_{graphite} + m_{StageIII}(1 - f_{StageIII}) + m_{StageII}(1 - f_{StageII}) + m_{StageI}(1 - f_{StageI})}{MW_C}}\right) \quad S1$$

Current and current density estimation from XRD data

The current necessary to change the electrode's lithiation state, x in Li_xC_6, was calculated using Faraday's constant (F):

$$I = \frac{\Delta x}{\Delta t}\frac{FM_{gr}}{MW_{C6}} \tag{S2}$$

The current density values for the plots shown in the main manuscript were determined by calculating the current for position (I_{local}) and dividing by the surface area of graphite estimated to be within the region of measurement.

$$I_{density} = \frac{I_{local}}{A_{sp.gr}M_{gr.local}} \tag{S3}$$

This estimation assumes that each region of measurement contains the same specific surface area and mass of graphite, i.e. homogeneous material properties and distribution of graphite within the electrode.

3. P3D model documentation

A Pseudo-3D model (P3D) model was developed in COMSOL Multiphysics. The model parameters were mainly taken from Colclasure et al.[2] and are reported in Table S1. Model parameters that depend on either Li concentration or temperature are reported in Table S2. The open-circuit potential and the exchange current density for both electrodes have quite complicated functional forms and are not repeated here (see Colclasure et al.[2]). The modeled equations use the native governing equations

implemented in COMSOL's Battery Design Module. These equations are not repeated here, but the reader is directed to COMSOL's user guide for the complete set of equations[3].

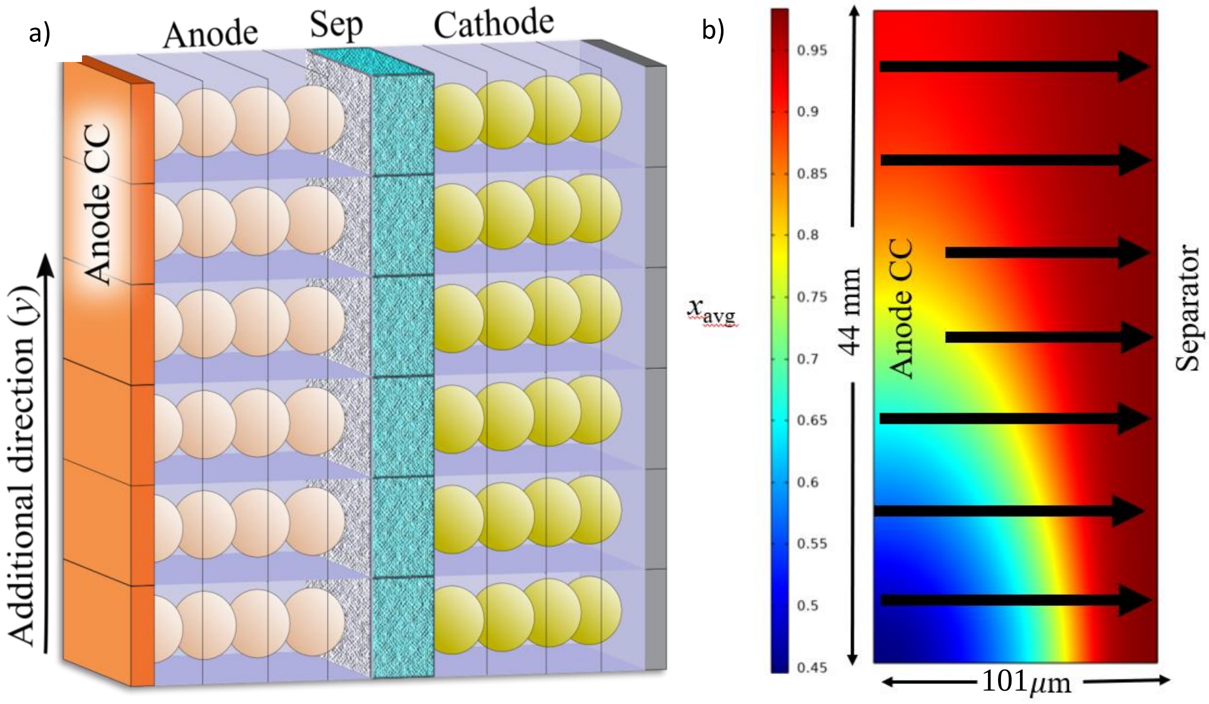

Figure S5: (a) Graphical representation of pseudo-3D model developed in COMSOL Multiphysics. The additional direction added to the standard pseudo-2D model is indicated as *y*. (b) Example of projecting the average intercalation fraction via integration to reproduce XRD data measured in-plane intercalation fraction distribution.

Figure S5a illustrates a model domain graphic. As illustrated an additional direction (*y*) is added to a standard pseudo-2D model to incorporate in-plane electrolyte heterogeneities, resulting in a P3D model. In this P3D model, the electrode potential, electrolyte Li-ion concentration, and electrolyte potential are solved both in-plane (*y*) and through-plane (*x*) directions. The solid-phase Li-concentration is solved in a third radial coordinate located in each (*x*,*y*) mesh node in either electrode.

Figure S5b illustrates the linear projection method used to extract averaged through-plane variables (see Figure 7 in main manuscript). The x-y contour plot illustrates the average intercalation fraction, which is extracted from the radial mesh as:

$$\mathrm{x_{avg}} = \frac{3}{R^3}\int_0^{\mathrm{R}} \mathrm{r}^2 \mathrm{x_{Li}}\ \mathrm{dr},$$

where *R* is the representative anode particle radius, x_{Li} is the local intercalation fraction, and x_{avg} is the average intercalation fraction within a particle. The XRD measurement averages the graphite lithiation

signal in the through-plane direction. A linear projection is used on the radially averaged intercalation fraction as:

$$x_{\mathrm{avg,proj}}(y) = \frac{1}{t_{\mathrm{an}}}\int_0^{\mathrm{t_{an}}} x_{\mathrm{avg}}(x,y)dx,$$

where t_{an} is the anode thickness and $x_{avg,proj}$ is the intercalation signal that would be measured in the XRD. The maximum, minimum, and mean $x_{avg,proj}$ is plotted on Figure 7 in the main manuscript at each timestep.

The current density in-plane projection (Figure 8 in manuscript) is handled in a similar way and can be represented as:

$$\mathrm{i}_{\mathrm{avg,\ in\text{-}plane}}(y) = \frac{1}{t_{\mathrm{an}}}\int_0^{\mathrm{t_{an}}} i(x,y)dx,$$

where *i* is the local current density and $i_{avg,in\text{-}plane}$ is the averaged current density. The maximum, minimum, and mean averaged current density is plotted in Figure 8 in the main manuscript. The through-plane averaging (Figure 8b) is handled mathematically as:

$$\mathrm{i}_{\mathrm{avg,\ through\text{-}plane}}(x) = \frac{1}{h_{\mathrm{bat}}}\int_0^{h_{\mathrm{bat}}} i(x,y)dy,$$

where h_{bat} is the in-plane battery height.

2.1 **Modeling heterogeneous in-plane Li-ion transport**

The in-plane direction is assumed to have heterogeneous Li-ion transport informed by MacMullin distributions measured by Liu et al.[1] Liu et al. measured the MacMullin number distribution across electrode sheets (a 2D surface). To reduce the 2D data into a single in-plane direction for the P3D model, a representative line-scan is taken from the 2D data to produce a MacMullin distribution as a function of *y*-direction only. Figure S6 illustrates the MacMullin number data points and nineth-order polynomial fit that is implemented in the P3D model. The MacMullin number can be represented as a ratio of tortuosity and porosity as:

$$\mathrm{N_m} = \frac{D_{\mathrm{bulk}}}{D_{\mathrm{eff}}} = \frac{\tau^2}{\epsilon_{\mathrm{el}}},$$

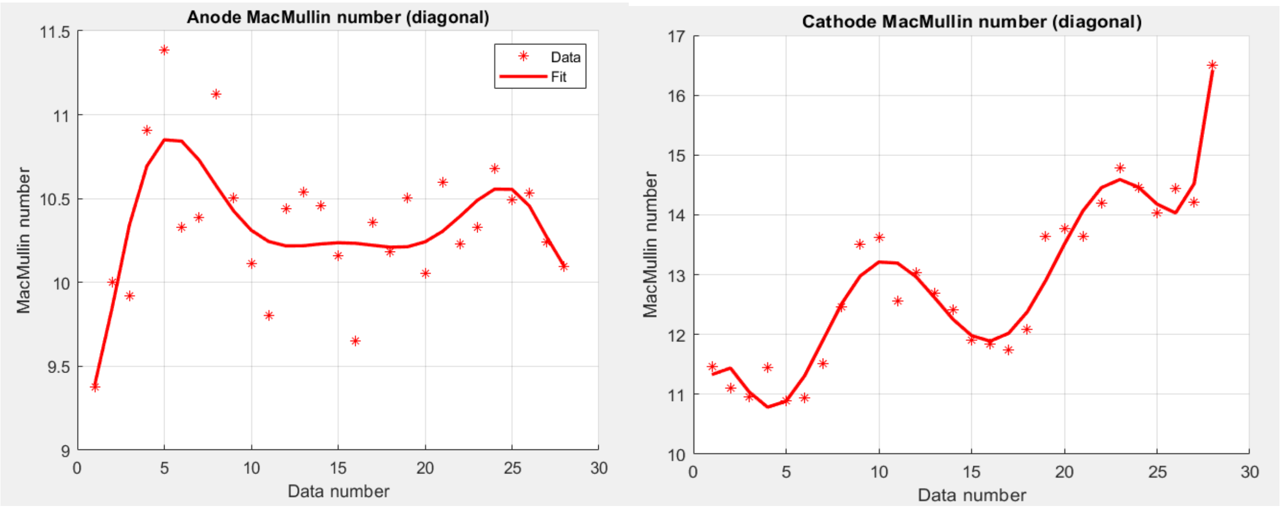

Figure S6: MacMullin number data measured by Liu et al.[1] and nineth-order polynomial fit implemented in the P3D model for the (a) anode and (b) cathode.

where N_m is the MacMullin number, D_{bulk} is the bulk diffusion, D_{eff} is the effective diffusion through the porous media, τ is the electrolyte tortuosity and ε_{el} is the electrolyte volume fraction. For the present study, the active loading is assumed to be uniform (i.e., ε_{el} is a constant) and the tortuosity is assumed to vary spatially along the *y*-direction (i.e., solve for τ using the N_m distribution plotted in Figure S4). As illustrated, the MacMullin distributions are significantly larger in the cathode than in the anode.

Figure S7 illustrates a representative intercalation fraction distribution simulated in the model. Figure S7a illuastrates the anode MacMullin distribution assumed in the additional in-plane direction. Similarly, Figure S7c illustrates the cathode MacMullin distribution assumed in the additional in-plane direction. Figures S7b illustrates contour plots of the average intercalation fraction x_{avg} after 900 s into the 6C CC-CV charge. Note that the verticle axis is on the order of 10s of mm while the horizontal axis is on the order of 100s of µm. This disparate scaling is implemented to clearly visualize the distributions. As illustrated the anode in-plane intercalation fraction distribution is small (i.e., the colors don't change much vertically). However, the through-plane distribution is quite large (i.e., the colors change significantly horizontally). This indicates that the Li-ion through-plane resistances are large to percolate through the porous electrode, while the in-plane resistances are relatively smaller. The cathode has more through-plane Li variance in the in-plane (verticle) direction than the anode. This increased in-plane dependence is because the cathode MacMullin number distribution is significantly higher than the anode MacMullin number distribution (compare Figure S7a and Figure S7c). However, even with increased MacMullin number ranges, the model predicts that the through-plane (horizontal) Li distribution is significantly higher than the in-plane (verticle) distribution. Again, this illustrates that the

model predicts significant Li transport resistances through the porous electrodes as opposed to along the porous electrode.

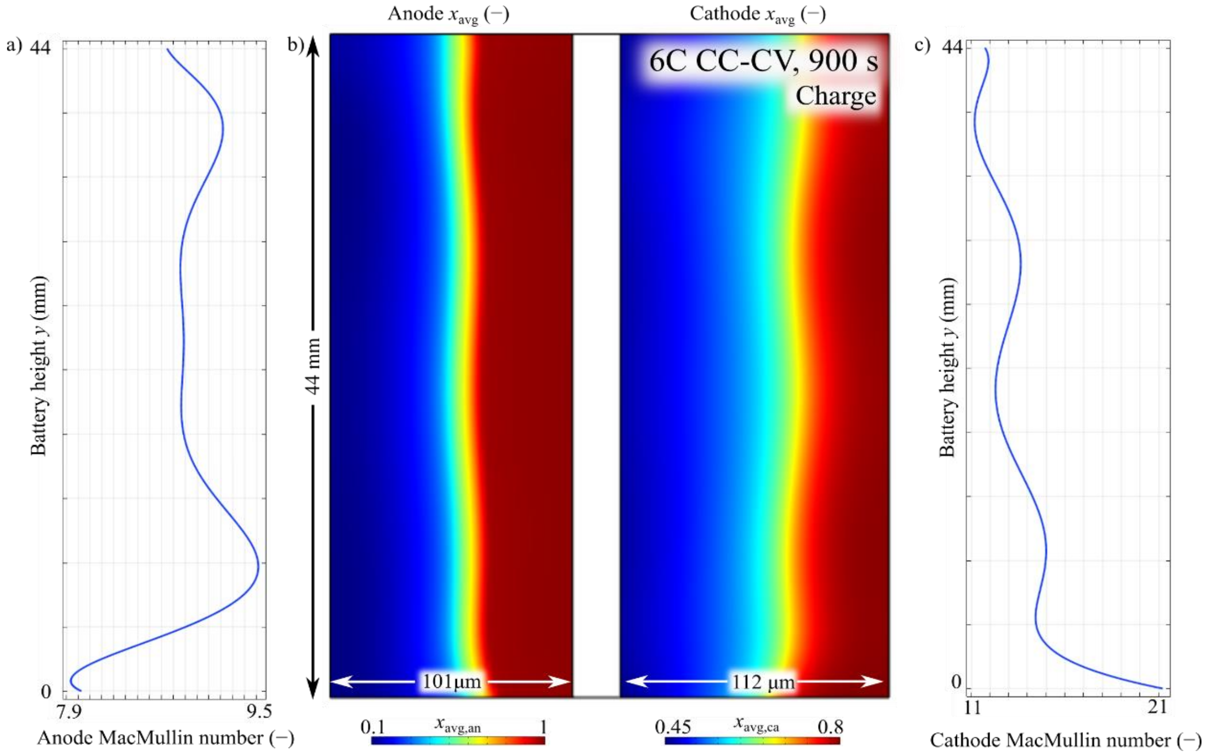

Figure S7: (a) Anode MacMullin number distribution along the battery height. (b) Contour plots of either electrode 900 s into the 6C CC-CV charge. (c) Cathode MacMullin number distribution along the battery height.

Table S1: Model parameters

Variable	Description	Value	Unit
h_{bat}	Battery height (in-plane direction)	44	mm
$c_{s,an,max}$	Anode maximum Li concentration	30	kmol m^{-3}
$x_{an,0}$ (charge)	Anode starting intercalation fraction (charge)	0.02	-
$x_{an,0}$ (discharge)	Anode starting intercalation fraction (discharge)	0.95	-
$c_{s,ca,max}$	Cathode maximum Li concentration	62	kmol m^{-3}
$x_{ca,0}$ (charge)	Cathode starting intercalation fraction (charge)	0.865	-
$x_{ca,0}$ (discharge)	Cathode starting intercalation fraction (discharge)	0.45	-
$c_{el,0}$	Electrolyte initial Li-ion concentration	1.2	kmol m^{-3}
t_{an}	Anode thickness	101	µm
t_{sep}	Separator thickness	20	µm
t_{ca}	Cathode thickness	112	µm
$R_{s,an}$	Anode representative particle radius	8.47	µm
$R_{s,ca}$	Cathode representative particle radius	8.47	µm
T	Temperature	308.15	K
α	Anodic and cathodic transfer coefficient	0.5	-
$\varepsilon_{el,an}$	Anode electrolyte volume fraction	0.362	-
$\varepsilon_{el,sep}$	Separator electrolyte volume fraction	0.40	-
$\varepsilon_{el,ca}$	Cathode electrolyte volume fraction	0.34	-
$\varepsilon_{s,an}$	Anode active material volume fraction	0.576	-
$\varepsilon_{s,an}$	Cathode active material volume fraction	0.524	-
$\sigma_{s,an}$	Anode solid-phase electrical conductivity	6.38	S m^{-1}
$\sigma_{s,ca}$	Cathode solid-phase electrical conductivity	6.6	S m^{-1}

Table S2: Model parameters with functional dependence[2].

Variable	Description	Function	Unit
D_{an}	Anode diffusion coefficient	$D_{\mathrm{an}}(T) = 3\mathrm{E}{-}13 \exp\left(\frac{-30\mathrm{E}6}{R}\left(\frac{1}{T} - \frac{1}{303.15}\right)\right)$	$\mathrm{m}^2\ \mathrm{s}^{-1}$
D_{ca}	Cathode diffusion coefficient	$D_{\mathrm{s,ca}}(x,T) = 1260 \times 10.0^{f(x)} \exp\left(\frac{-30\mathrm{E}{+}6}{R} * \left(\frac{1}{T} - \frac{1}{303.15}\right)\right),$ $f(x) = -2.509010843479270\mathrm{E}{+}2x^{10} + 2.391026725259970\mathrm{E}{+}3x^9 - 4.868420267611360\mathrm{E}{+}3x^8 - 8.331104102921070\mathrm{E}{+}1x^7 + 1.057636028329000\mathrm{E}{+}4x^6 - 1.268324548348120\mathrm{E}{+}4x^5 + 5.016272167775530\mathrm{E}{+}3x^4 + 9.824896659649480\mathrm{E}{+}2x^3 - 1.502439339070900\mathrm{E}{+}3x^2 + 4.723709304247700\mathrm{E}{+}2x - 6.526092046397090\mathrm{E}{+}1$	$\mathrm{m}^2\ \mathrm{s}^{-1}$
D_{Li^+}	Li-ion diffusion coefficient	$\log_{10}\left[D_{\mathrm{e}}(c_{\mathrm{e}},T)\right] = 1\mathrm{E}{-}4\left(-0.5688226 - \frac{1607.003}{T-(-24.83763+64.07366c_{\mathrm{e}})} + \left(-0.8108721 + \frac{475.291}{T-(-24.83763+64.07366c_{\mathrm{c}})}\right)c_{\mathrm{e}} + \left(-0.005192312 - \frac{33.43827}{T-(-24.83763+64.07366c_{\mathrm{e}})}\right)c_{\mathrm{e}}^2\right)$	$\mathrm{m}^2\ \mathrm{s}^{-1}$
κ_{Li^+}	Ionic conductivity	$\kappa_{\mathrm{e}}(c_{\mathrm{e}},T) = c_{\mathrm{e}}\Big((0.0001909446T^2 - 0.08038545 * T + 9.00341) + (-0.00000002887587T^4 + 0.00003483638T^3 - 0.01583677T^2 + 3.195295T - 241.4638) * c_{\mathrm{c}} + (0.00000001653786T^4 - 0.0000199876T^3 + 0.009071155 * T^2 - 1.828064 * T + 138.0976)c_{\mathrm{c}}^2 + (-0.000000002791965T^4 + 0.000003377143T^3 - 0.001532707T^2 + 0.3090003T - 23.35671)c_{\mathrm{c}}^3\Big)$	$\mathrm{S\ m}^{-1}$
$\frac{\mathrm{d}\ln f_\pm}{\mathrm{d}\ln c_{\mathrm{e}}}$	Activity coefficient	$\frac{\mathrm{d}\ln f_\pm}{\mathrm{d}\ln c_{\mathrm{e}}} = 0.54c_{\mathrm{e}}^2 \exp\left(\frac{329.0}{T}\right) - 0.00225c_{\mathrm{e}} \exp\left(\frac{1360.0}{T}\right) + 0.341 \exp\left(\frac{261.0}{T}\right)$	–
t_+^0	Transference number	$t_+^0 = (-0.0000002876102T^2 + 0.0002077407T - 0.03881203)c_{\mathrm{e}}^2 + (0.000001161463T^2 - 0.00086825T + 0.1777266)c_{\mathrm{e}} + (-0.0000006766258T^2 + 0.0006389189T + 0.3091761)$	–

R is the universal gas constant in units of J $\mathrm{kmol}^{-1}\ \mathrm{K}^{-1}$, x is the intercalation fraction, T is temperature in units of [K], and c_{e} is Li-ion concentration in units of kmol m^{-3}

References

1. Liu, B.; Prugue, K.; Nikpour, M.; Ward, K.; Mazzeo, B. A.; Wheeler, D. R., Heterogeneity in MacMullin Number of Li-Ion Battery Electrodes Studied by Means of an Aperture Probe. *Journal of The Electrochemical Society* **2022,** *169* (1), 010517.
2. Colclasure, A. M.; Dunlop, A. R.; Trask, S. E.; Polzin, B. J.; Jansen, A. N.; Smith, K., Requirements for Enabling Extreme Fast Charging of High Energy Density Li-Ion Cells while Avoiding Lithium Plating. *Journal of The Electrochemical Society* **2019,** *166* (8), A1412-A1424.
3. *COMSOL Battery Design Module User's Guide*; COMSOL: 2020.

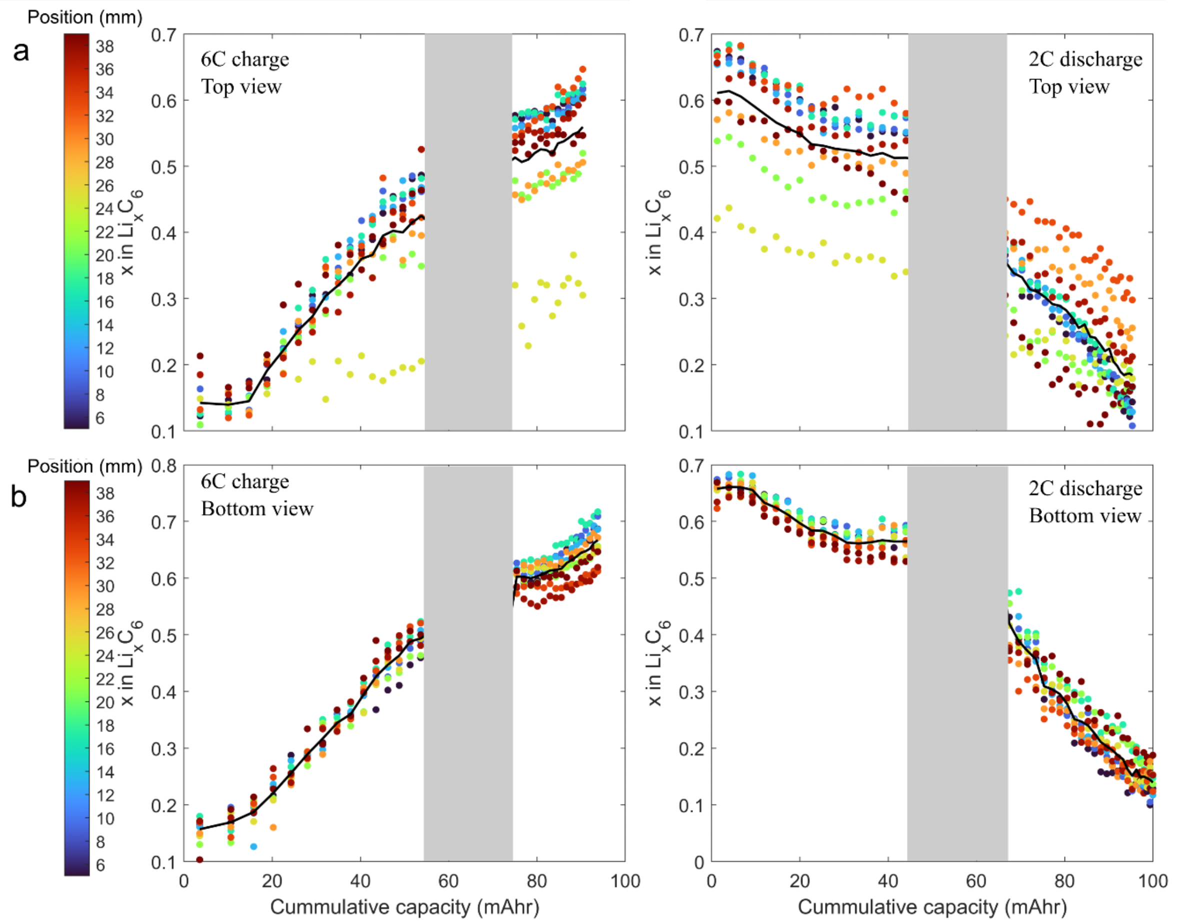

Figure S8: The state of lithiation x in Li_xC_6 spatially and temporally measured within the pouch cell during 6C charge and 2C discharge measured at (a) the top view and (b) the bottom view with respect to charge/discharge cumulative capacity. Illustrated are the same data as in Figure 2 in the main manuscript, but here the data is plotted with respect to capacity as opposed to with respect to time.
